# Proteomics Unravels the Regulatory Mechanisms in Human Tears Following Acute Renouncement of Contact Lens Use: A Comparison between Hard and Soft Lenses

**DOI:** 10.1038/s41598-018-30032-5

**Published:** 2018-08-01

**Authors:** Caroline Manicam, Natarajan Perumal, Joanna Wasielica-Poslednik, Yong Cajetan Ngongkole, Alexandra Tschäbunin, Marcel Sievers, Walter Lisch, Norbert Pfeiffer, Franz H. Grus, Adrian Gericke

**Affiliations:** Department of Ophthalmology, University Medical Centre of the Johannes Gutenberg University Mainz, 55131 Mainz, Germany

## Abstract

Contact lenses (CLs) provide a superior alternative to spectacles. Although beneficial, the global burden of ocular dysfunctions attributed to regular use of CLs remains a topic of much challenge in ophthalmic research owing to debilitating clinical repercussions on the ocular surface, which are often manifested as breach in tear film integrity. This study elucidated the intricate tear proteome changes attributed to the use of different CLs (hard and soft) and unravelled, for the first time, the restorative mechanisms of several protein clusters following acute renouncement of CL use employing the label-free mass spectrometry-based quantitative proteomics approach. The expression patterns of certain proteins clusters were specific to the use of a particular lens type and a large majority of these actively regulates cell death and survival and, modulates cellular movement on the ocular surface. Noteworthy, CL use also evoked a significant upregulation of glycolytic enzymes associated with hypoxia and corresponding cognate metabolic pathways, particularly glucose metabolism and FXR/RXR pathways. Importantly, the assessment of CL renouncement unravelled the restorative properties of several clusters of proteins involved mainly in organismal injury and abnormalities and, cellular function and maintenance. These proteins play key roles in restoring tear homeostasis and wound-healing mechanisms post-CL use-elicited injury.

## Introduction

The advent of contact lenses (CLs) has dramatically revolutionized the eye care system by providing a superior alternative to the use of spectacles and has gained popularity over the years owing to several factors ranging from convenience of wear, achievement of a better vision, as well as cosmesis^1,2^. Currently, there are approximately 14 million CL wearers worldwide and this number is growing steadily every year^3,4^. Albeit fascinating technological advancements in improving the quality and biocompatibility of the lenses over the years, CLs are still foreign objects on the ocular surface, which alter tear homeostasis by disrupting tear film stability and changing the levels of tear molecular constituents^5,6^. It is, therefore, not surprising that CL-induced inflammation is one of the major causes of morbidity in the United States of America^7^. Great strides have been made in the past decades to better understand and address the pathophysiology of the complications underlying CL wear at both biophysical and biochemical levels^8–11^.

It is well-recognized that even a slight shift in the ocular physiological state can trigger germane changes in the tear proteome and be readily detected with high sensitivity utilizing several sophisticated laboratory techniques, as demonstrated previously, including some of our preceding studies in reflex tears and dry eye syndrome^12–14^. Our previous investigations have also shown that there are distinct changes in the tear composition of CL users compared to non-users and, the use of different lens types influenced the protein profiles^15,16^. However, to date, there is still an unmet need for extensive characterization of the adverse changes that take place on the ocular surface, particularly in the tear fluid proteome, associated with the use of CLs to better understand the molecular mechanisms underlying potential pathogenesis of CL-related ocular disorders. Moreover, it remains to be unraveled if the renouncement of CL use, albeit for a short period of time, can have favorable restorative effects on the protein regulation profiles on the ocular surface.

**Figure 1:**
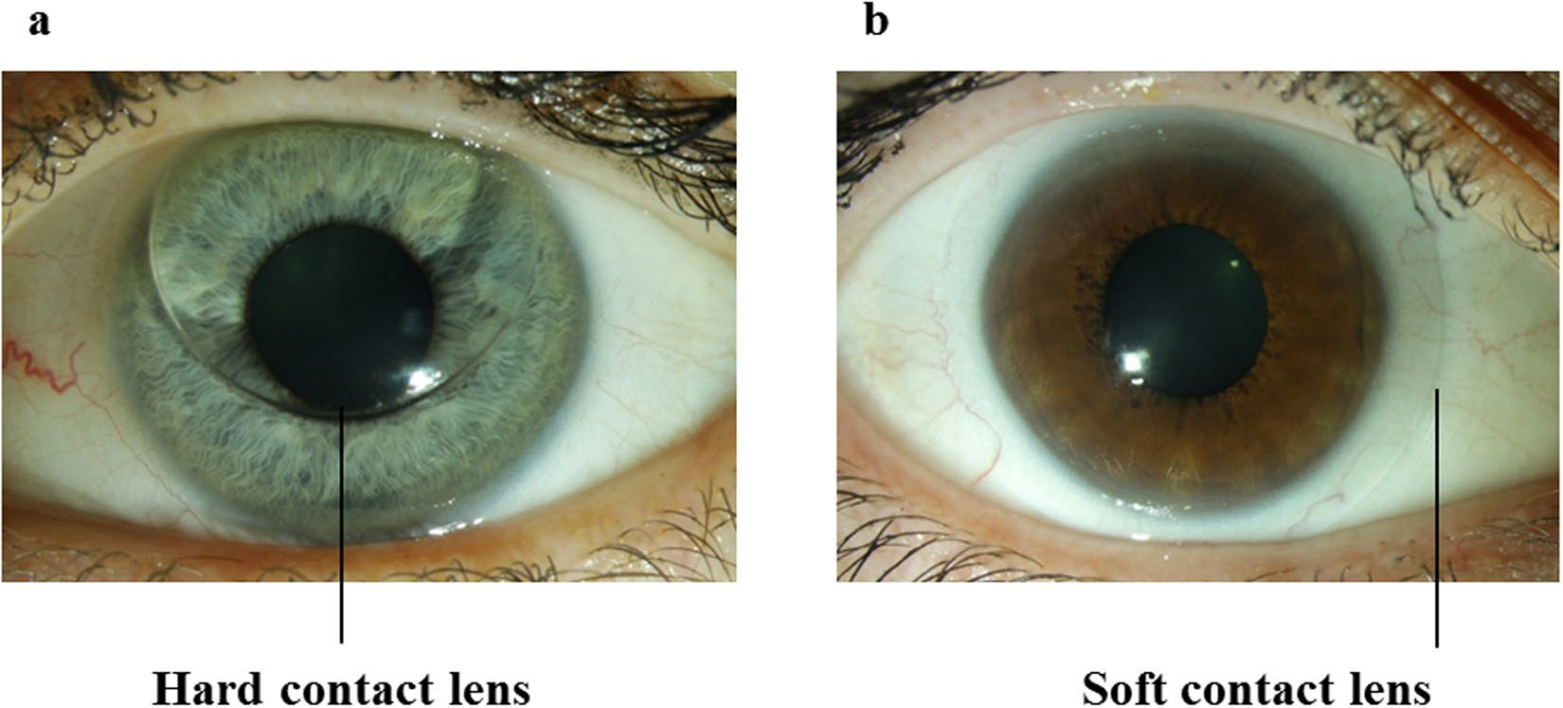
Two major types of contact lenses. Photographs showing the (**a**) hard and (**b**) soft contact lenses on the ocular surface.

Therefore, considering the high prevalence of CL users worldwide and the paucity of in-depth insight into the mechanistic alterations at the protein level attributed to the use of two main types of CLs (rigid gas permeable or hard and soft CLs; Fig. 1), this study elucidated the intricate molecular changes in the tear protein constituents employing the state-of-the-art mass spectrometry-based proteomics platform and bioinformatics tools. Importantly, this investigation has also provided the first systematic outlook into the dynamic protein profiles of CL users following an acute renouncement period of lens usage.

## Results

### Quantitative Tear Proteomics of CL Users

**Figure 2:**
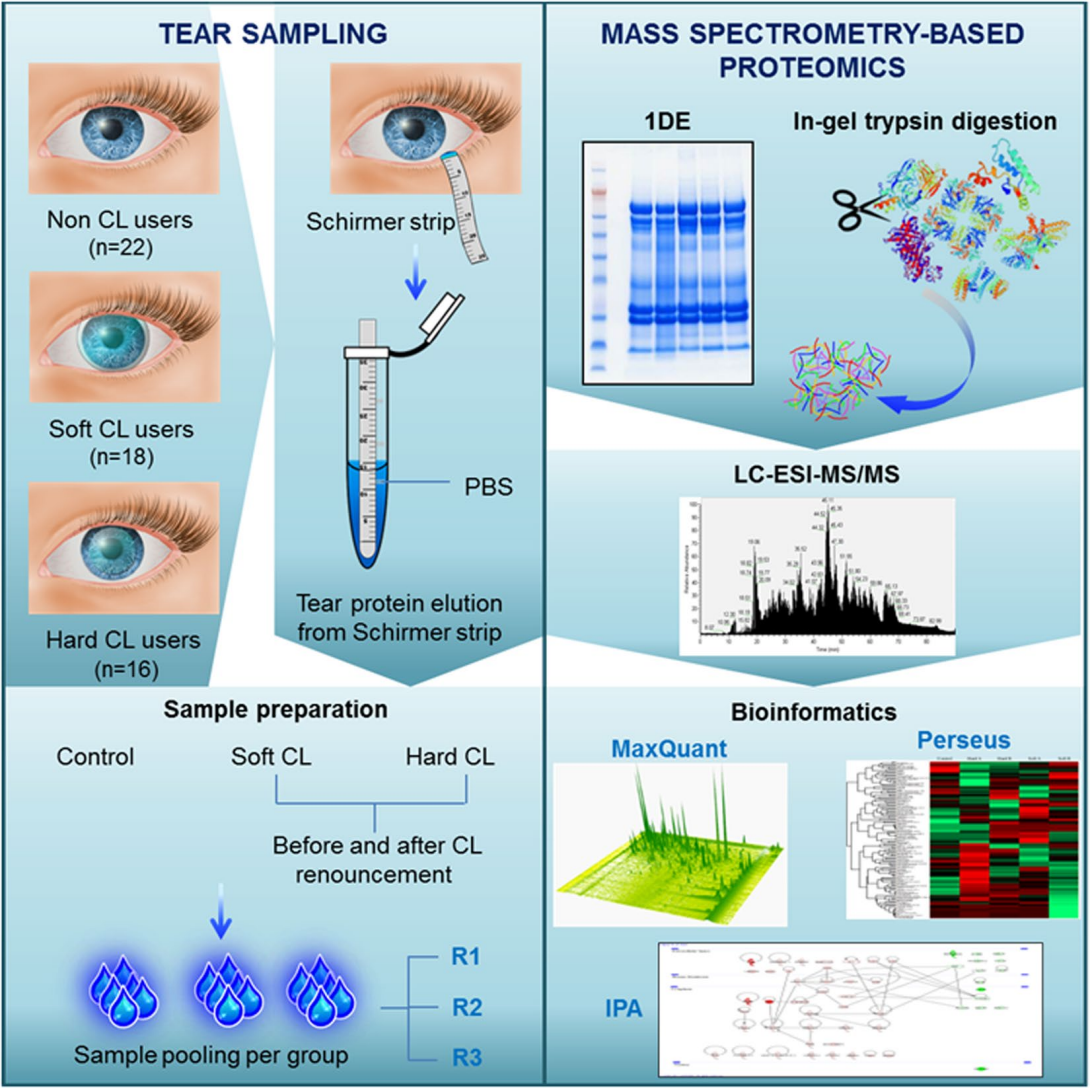
Workflow overview. Tear samples were collected with Schirmer strips from soft and hard CL users before and after renouncement of CL use. Tears from non-CL users are designated as controls. Samples from the respective groups were pooled after protein measurements and subjected to 1DE gel electrophoresis, trypsin-digestion and bottom-up proteomics analyses employing LC-ESI-MS/MS. The emerging continuum MS datasets were subjected to robust bioinformatics analyses and functional annotations using various tools comprising the MaxQuant computational proteomics platform^74,75^, Perseus^76^ and IPA software (QIAGEN Inc., https://www.qiagenbioinformatics.com/products/ingenuity-pathway-analysis)^78^ to identify the differential protein expressions and protein interaction networks.

**Figure 3:**
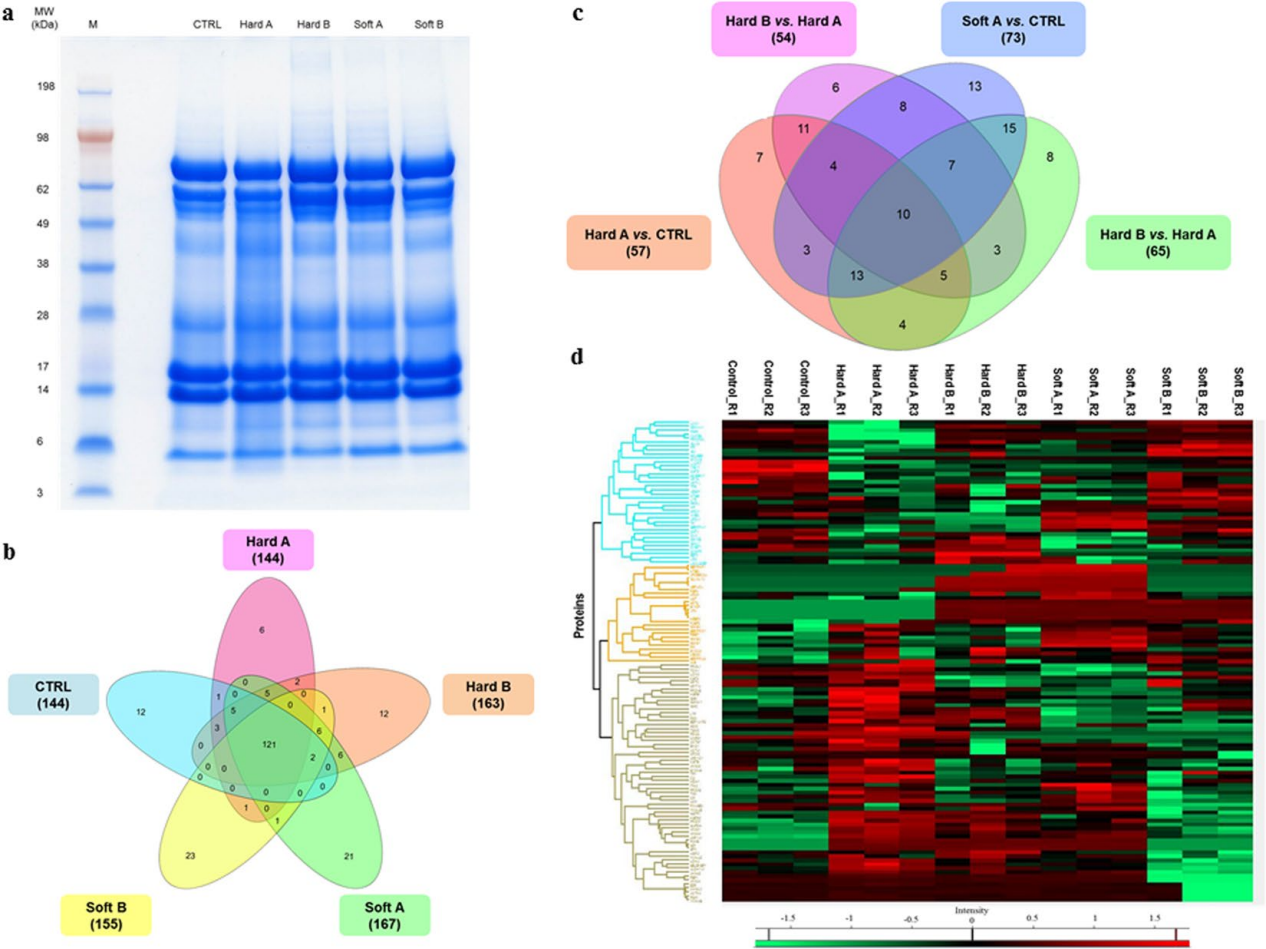
Tear proteome of hard and soft CL users. (**a**) Representative tear protein profiles of both types of CL wearers before (designated as A) and after (designated as B) CL renouncement compared to non-CL users (designated as CTRL) resolved in 1DE gel stained with colloidal blue. M: marker. (**b**) Venn diagram depicting overlaps of identified tear proteins in hard and soft CL users before and following lens renouncement compared to the control group. (**c**) Venn diagram showing overlaps of differentially expressed tear proteins in each CL subgroup before and after CL renouncement. (**d**) Heat map depicts the hierarchical clustering of the differentially expressed tear proteins in the hard and soft CL groups before (designated as A) and after (designated as B) CL renouncement compared to the CTRL.

Bottom-up MS-based discovery proteomics approach was employed in the present study to identify and distinguish the tear proteome profiles of two major groups of CL wearers. The overview of the tear sampling and proteomics workflow employed in this study is depicted in Fig. 2. Representative tear protein profiles of respective CL users before (designated as A) and after the discontinuation (designated as B) of CL use compared to non-CL wearers (designated as CTRL) resolved in first dimensional gel electrophoresis (1DE) are depicted in Fig. 3a. A total of 230 proteins were identified by label-free quantification (LFQ) at a false discovery rate (FDR) of 1% (the complete list of proteins can be found as Supplementary Table S2). The use of soft CLs resulted in the expression of a slightly higher number of proteins (167 proteins) than the hard CLs (144 proteins), with an overlap of 131 proteins between both groups (Fig. 3b). There were a number of proteins that were exclusively observed in each subgroup, with 12 proteins in the CTRL and hard B groups, 6 proteins in hard A, 21 and 23 proteins in soft A and soft B, respectively.

### Differential Expression of Proteins in Tears of Soft and Hard CL Users

Next, the LFQ values obtained from MaxQuant analysis were used for statistical analysis employing Perseus software to identify the differentially expressed proteins in both soft and hard CL users. In general, the label free quantifications was reproducible across all samples and demonstrated excellent technical reproducibility, as shown by high *R* values of 0.96 ± 0.02 for hard A and, 0.99 ± 0.0 for control, hard B, soft A and soft B. On average, correlation between most of the designated groups were > 0.95, and slightly lower correlations were observed between the soft A *vs*. hard A (0.91 ± 0.05) and soft B *vs*. hard A (0.89 ± 0.05) groups. These findings demonstrated that there are high similarities between the proteome of the designated groups and this analysis also indicates that reproducible data were generated from the pooled tear samples, which enabled further statistical analysis (Supplementary Table S3).

The general overview of the number of proteins identified to be significantly differentially expressed in each subgroup before and after discontinuation of CL use is represented in the Venn diagram in Fig. 3c. Thirty similar proteins were found to be significantly differentially expressed (p < 0.05) in the tear samples of both hard and soft CL users compared to the CTRL group, as listed in Table 1. Among these protein markers, 9 were decreased in abundance and 18 were increased in both hard and soft CL subgroups. Cystatin D (CST5) was the most significantly down-regulated protein, while protein disulfide-isomerase (P4HB), 14-3-3 protein sigma (SFN) and glucose-6-phosphate isomerase (GPI) were drastically up-regulated in both CL users. A comparison between the use of hard and soft CLs demonstrated that 58 proteins were differentially expressed between these groups, with 36 significantly up- and 22 down-regulated proteins (Table 2). The five proteins that were most significantly decreased in abundance in the hard CL compared to soft CL users comprised keratin, type II cytoskeletal 5 (KRT5), DNA-directed primase/polymerase protein (PRIMPOL), vitronectin (VTN), nucleobindin-2 (NUCB2) and Ig lambda chain V-IV region Hil (IGLV3-10). On the other hand, the proteins that were significantly increased in abundance composed of UMP-CMP kinase (CMPK1), protein disulfide-isomerase A3 (PDIA3), peroxiredoxin-6 (PRDX6), pyruvate kinase (PKM) and cystatin-C (CST3).

**Table 1:** List of the significantly differentially expressed tear proteins identified in both hard and soft contact lens users.

Gene name	Hard A/CTRL			Soft A/CTRL		
	*P-* value	Log_2_ ratio	Abundance	*P-* value	Log_2_ ratio	Abundance
SFN	1.8E − 07	2.2E + 01	High	1.3E − 08	2.2E + 01	High
GPI	3.4E − 07	2.2E + 01	High	8.6E − 11	2.2E + 01	High
P4HB	1.3E − 05	2.1E + 01	High	6.3E − 09	2.1E + 01	High
FBP1	1.4E − 04	2.5E + 00	High	4.0E − 04	2.5E + 00	High
AKR1A1	1.1E − 05	2.0E + 00	High	1.4E − 04	2.0E + 00	High
GAPDH	3.7E − 05	1.8E + 00	High	4.9E − 03	1.8E + 00	High
ANXA2	1.1E − 04	1.7E + 00	High	1.0E − 04	1.7E + 00	High
ANXA1	3.4E − 04	1.7E + 00	High	3.9E − 03	1.7E + 00	High
GSTP1	1.2E − 04	7.6E − 01	High	2.6E − 04	7.6E − 01	High
C3	2.2E − 04	5.8E − 01	High	3.3E − 02	5.8E − 01	High
TPI1	4.5E − 02	4.1E − 02	High	4.1E − 02	5.6E − 01	High
PRDX1	4.9E − 03	3.9E − 02	High	3.9E − 02	8.1E − 01	High
SERPINC1	4.9E − 02	3.4E − 02	High	3.4E − 02	6.9E − 01	High
GSN	2.0E − 02	2.0E − 02	High	2.0E − 02	1.3E + 00	High
CTSB	2.5E − 03	1.9E − 02	High	1.9E − 02	−5.9E − 01	Low
A2M	1.9E − 03	1.1E − 02	High	1.1E − 02	1.4E + 00	High
PKM	5.7E − 03	6.9E − 03	High	6.9E − 03	1.3E + 00	High
TGM2	9.2E − 03	6.7E − 03	High	6.7E − 03	1.3E + 00	High
ZG16B	6.9E − 03	5.4E − 03	High	5.4E − 03	−6.4E − 01	Low
PRDX5	1.4E − 02	4.7E − 03	High	4.7E − 03	1.1E + 00	High
PROL1	2.4E − 02	3.9E − 03	High	3.9E − 03	7.1E − 01	High
AZGP1	3.1E − 02	2.5E − 03	High	2.5E − 03	−9.2E − 01	Low
SCGB2A1	4.1E − 02	1.3E − 03	High	1.3E − 03	−8.1E − 01	Low
MSLN	6.4E − 03	5.6E − 04	High	5.6E − 04	−9.3E − 01	Low
PEBP1	2.1E − 02	8.2E − 05	High	8.2E − 05	6.3E − 01	High
TF	4.9E − 02	1.3E − 05	High	1.3E − 05	4.9E − 01	High
PIGR	7.4E − 04	−1.0E + 00	Low	3.5E − 03	−1.0E + 00	Low
LACRT	9.6E − 06	−3.3E + 00	Low	6.9E − 05	−3.3E + 00	Low
CST5	1.1E − 10	−2.1E + 01	Low	1.1E − 10	−2.1E + 01	Low
NUCB2	6.7E − 08	−2.3E + 01	Low	1.2E − 02	−2.3E + 01	Low

**Table 2:** List of the significantly differentially expressed tear proteins in hard compared to soft contact lens users.

Gene name	Hard A/Soft A		
	*P-* value	Log_2_ ratio	Abundance
KRT5	2.90E − 11	−1.91E + 01	Low
PRIMPOL	1.38E − 09	−2.21E + 01	Low
VTN	3.51E − 09	−2.07E + 01	Low
NUCB2	8.15E − 09	−2.19E + 01	Low
IGLV3-10	1.75E − 08	−2.07E + 01	Low
SERPINF1	3.49E − 08	−1.82E + 01	Low
S100P	9.51E − 08	−2.15E + 01	Low
APOBEC3A	9.82E − 08	−2.03E + 01	Low
HEBP2	1.39E − 07	−2.15E + 01	Low
CMPK1	1.63E − 07	2.17E + 01	High
ABRACL	1.77E − 07	−1.97E + 01	Low
PDIA3	1.82E − 07	2.14E + 01	High
CTSD	4.27E − 07	−2.08E + 01	Low
LACRT	1.11E − 05	−1.19E + 00	Low
PRDX6	6.52E − 05	3.11E + 00	High
PKM	1.11E − 04	2.41E + 00	High
CST3	2.26E − 04	9.07E − 01	High
ALDH1A1	7.43E − 04	9.06E − 01	High
TGM2	8.89E − 04	2.11E + 00	High
C3	1.06E − 03	4.11E − 01	High
PROL1	1.41E − 03	1.60E + 00	High
SERPINA1	1.51E − 03	−9.13E − 01	Low
TCN1	1.90E − 03	1.85E + 00	High
ANXA1	2.28E − 03	7.73E − 01	High
LDHA	2.46E − 03	1.05E + 00	High
GSTP1	2.49E − 03	3.79E − 01	High
LCN2	2.96E − 03	8.09E − 01	High
GC	3.53E − 03	−7.63E − 01	Low
APOA1	3.65E − 03	−1.34E + 00	Low
S100A9	4.13E − 03	6.77E − 01	High
IGKV4-1	4.61E − 03	8.63E − 01	High
HSPB1	4.93E − 03	1.15E + 00	High
GAPDH	5.14E − 03	8.33E − 01	High
MDH1	5.41E − 03	4.30E − 01	High
IGHA1	5.58E − 03	8.03E − 01	High
TF	5.65E − 03	−9.32E − 01	Low
PIGR	5.97E − 03	−5.01E − 01	Low
IGKC	6.08E − 03	1.09E + 00	High
A1BG	6.52E − 03	−1.31E + 00	Low
KRT2	6.64E − 03	1.19E + 00	High
EEF1A1P5	8.58E − 03	2.26E + 00	High
FBP1	9.89E − 03	8.72E − 01	High
APOH	1.10E − 02	−8.92E − 01	Low
ACTA1	1.20E − 02	9.69E − 01	High
SELENBP1	1.29E − 02	6.48E − 01	High
AGT	1.47E − 02	−1.23E + 00	Low
HSPG2	1.52E − 02	1.54E + 00	High
CST4	2.04E − 02	−6.33E − 01	Low
YWHAB	2.13E − 02	7.08E − 01	High
LTF	2.15E − 02	4.63E − 01	High
AKR1C1	2.17E − 02	5.67E − 01	High
EZR	2.73E − 02	1.47E + 00	High
CSTB	2.73E − 02	7.65E − 01	High
ACTG1	3.15E − 02	1.00E + 00	High
ACTN4	3.31E − 02	1.01E + 00	High
ITIH2	3.31E − 02	−1.03E + 00	Low
TTR	3.79E − 02	2.45E − 01	High
KRT10	4.99E − 02	9.16E − 01	High

**Figure 4:**
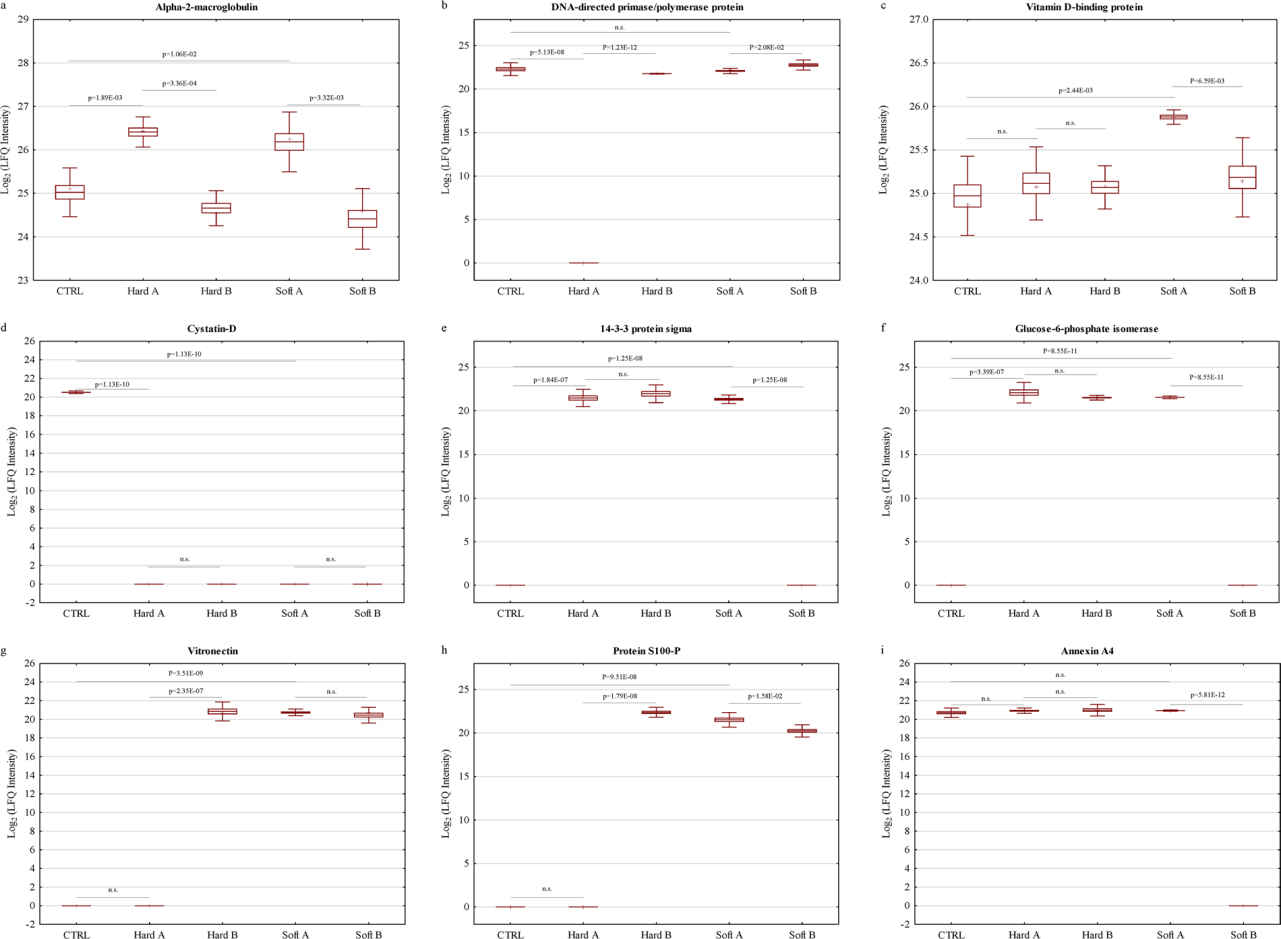
Differential expression profiles of exemplary tear proteins of hard and soft CL users compared to CTRL. Box plots showing the different expression profiles of some of the significantly (p < 0.05) differentially expressed tear proteins in the two CL users that were restored to near-normal after renouncement comprising (**a**) A2M (**b**) PRIMPOL and (**c**) GC. Some proteins were not restored to near-normal or normal levels following the discontinuation of CL use, which comprised (**d**) CST5 in both CL users, (**e**) SFN and (**f**) GPI in the hard CL users. The expression profiles of (**g**) VTN and (**h**) S100P show exemplary proteins that were only up-regulated after hard CL renouncement and, (**i**) ANXA4 was exclusively down-regulated in the soft CL following renouncement. The *y*-axis represents the log_2_ LFQ intensities of the proteins. Box represents the mean ± SE, whiskers represent mean ± 2*SD, plus sign (+) denotes the median and the horizontal line in the box denotes mean.

A heat map with unsupervised hierarchical clustering of the tear proteome in each CL subgroup and CTRL reveal the segregation of identified proteins into various clusters (Fig. 3d). Closer examination of the protein levels within each of the dominant clusters demonstrate three major phenomena that were observed in the present study following the renouncement of CLs for an acute period of time. First, a cluster of differentially expressed proteins was restored to near-normal or normal levels after CLs were ceased to be worn, which comprised 52.6% and 58.9% of the total differentially expressed proteins for hard and soft CLs, respectively (Supplementary Tables S4 and S5 for hard and soft CLs, respectively). However, a second cluster of proteins was not restored to normal levels, comprising 47.4% and 41.1% of the total differentially expressed proteins for hard and soft CLs, respectively. The lists of these non-restored protein clusters are tabulated in Supplementary Tables S6 and S7 for hard and soft CLs, respectively. The third cluster was characterized by the expression of certain proteins, which were only regulated after CL renouncement. In gist, the use of soft CLs elicited a higher alteration in tear protein expressions than its hard counterpart and, this group has also exhibited the highest recovery level following lens renouncement. Figure 4a depicts the regulation profiles of alpha-2-macroglobulin (A2M), an exemplary protein that was restored to near-normal level in tears of both CL users following renouncement. On the other hand, the differential expression of certain clusters of proteins was particular to a specific CL group, such as the DNA-directed primase/polymerase protein (PRIMPOL), which was found to be significantly reduced (p < 0.001) only in the hard CL users and its level was restored to normalcy after discontinuation of CL use (Fig. 4b). Similarly in the tears of soft CL wearers, one exemplary protein that was exclusively differentially expressed in this group was the vitamin D-binding protein (GC), which was restored to near-normal level following renouncement (Fig. 4c).

On the contrary, among the non-restored protein levels, CST5 was significantly down-regulated (p < 0.001) in both hard and soft CL users, and remained decreased even after the CLs were ceased to be worn, as shown in Fig. 4d. Interestingly, the regulation profiles of some proteins differ between the CL groups for the same marker. This is exemplified by the differential expressions of SFN and GPI in Fig. 4e and 4f, respectively. Both proteins were significantly upregulated in both hard and soft CL wearers, but their levels were restored to normal only after discontinuation of use of the soft CLs and remained elevated in their hard counterpart. Another striking finding in this study is that some proteins were only differentially expressed after the renouncement of CLs, such as the upregulation of vitronectin (VTN) and protein S100-P (S100P) in the tears of hard CL users (Fig. 4g and 4h, respectively), and the down-regulation of annexin A4 (ANXA4) in the soft CL users (Fig. 4i).

### Pathway Analysis of the Differentially Expressed Tear Proteins of CL users

To further unravel the functional and physiological significance of the differentially expressed tear proteins of the different types of CL users, we subjected our proteomics data to the Ingenuity Pathway Analysis (IPA) software to identify protein-protein interaction (PPI) networks, top canonical pathways and, molecular and cellular functions. The top three most significantly modulated pathways in the hard CL wearers comprised glycolysis, gluconeogenesis and FXR/RXR activation (Table 3). On the contrary, the use of soft CLs demonstrated the significant activation of three different pathways, which included LXR/RXR and FXR/RXR activations and, acute phase response signaling (Table 3). The canonical pathways of the differentially expressed proteins identified in hard and soft CLs represented as stacked bar chart are shown in Supplementary Figs S1 and S2, respectively. In this analysis, Fisher’s exact test p-values (p < 0.001) and a minimum of two molecules were used for scoring. The most significant molecular and cellular functions of the differentially expressed tear proteins of the hard CL wearers were involved in cell death and survival, cellular movement as well as in free radical scavenging (Table 4). Similarly in the soft CL users, the top molecular and cellular functions composed of cellular movement, free radical scavenging and, cell death and survival, as listed in Table 4.

**Table 3:** List of significantly modulated canonical pathways in tears of hard and soft CL users.

Canonical Pathways	−log (*P*-value)	Molecules
**Hard A** ***vs*** *.* **CTRL**		
Glycolysis I	8.31	GPI, TPI1, PKM, GAPDH, FBP1
Gluconeogenesis I	6.31	GPI, GAPDH, FBP1, MDH1
FXR/RXR activation	3.57	C3, TF, FBP1, A1BG
Aryl hydrocarbon receptor signaling	3.39	TGM2, ALDH1A1, GSTP1, HSPB1
Phagosome maturation	3.3	PRDX1, PRDX5, CTSB, PRDX6
Methylglyoxal degradation III	3.15	AKR1A1, AKR1C1/AKR1C2
NRF2-mediated oxidative stress response	2.87	AKR1A1, PRDX1, TXN, GSTP1
**Soft A** ***vs*** *.* **CTRL**		
LXR/RXR activation	21.2	TTR, C3, APOH, VTN, SERPINF1, ALB, LYZ, APOA1, TF, ORM1, ORM2, SERPINA1, GC, CLU, RBP4, AGT, TTR, C3, APOH, VTN, SERPINF1, ALB, APOA1, TF, ORM1, FBP1,
FXR/RXR activation	20.9	ORM2, SERPINA1, GC, CLU, RBP4, AGT
Acute phase response signaling	18.8	TTR, C3, APOH, SERPINF1, SERPINA3, ALB, HP, APOA1, ITIH2, TF, ORM1, ORM2, SERPINA1, A2M, RBP4, AGT
Glycolysis I	11.8	PGK1, ENO1, GPI, TPI1, PKM, GAPDH, FBP1
Clathrin-mediated endocytosis signaling	10.4	ALB, LYZ, APOA1, ORM1, TF, UBA52, ORM2, SERPINA1, ACTG1, CLU, RBP4
Atherosclerosis signaling	8.11	ALB, LYZ, APOA1, ORM1, ORM2, SERPINA1, CLU, RBP4
Gluconeogenesis I	7.72	PGK1, ENO1, GPI, GAPDH, FBP1

**Table 4:** Top molecular and cellular functions in tears of hard and soft CL users.

Molecular and Cellular Functions	*P*-value	# Molecules
**Hard A** ***vs.*** **CTRL**		
Cell Death and Survival	3.07E-03-2.32E-14	37
Cellular Movement	2.93E-03-3.28E-12	30
Free Radical Scavenging	2.48E-03-3.87E-12	17
Protein Trafficking	2.48E-03-1.41E-07	12
Small Molecule Biochemistry	3.03E-03-1.59E-07	28
**Soft A** ***vs.*** **CTRL**		
Cellular Movement	6.77E-04-5.48E-18 44	44
Free Radical Scavenging	4.55E-04-2.79E-12 22	22
Cell Death and Survival	7.11E-04-8.17E-12 47	47
Cell-To-Cell Signaling and Interaction	6.01E-04-1.30E-09 37	37
Cellular Development	7.01E-04-2.87E-09 38	38

**Figure 5:**
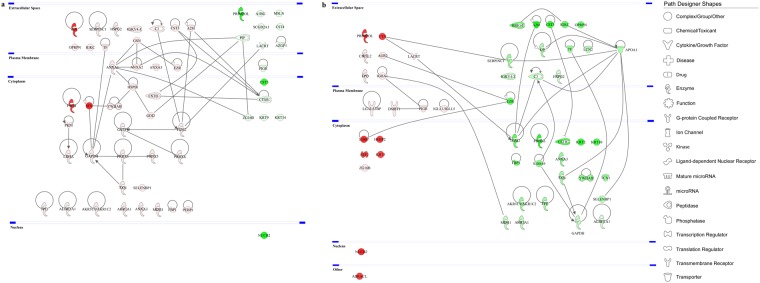
Protein-protein interaction networks of the differentially expressed tear proteins of hard CL users. The major interaction networks of differentially expressed tear proteins obtained by IPA analysis (QIAGEN Inc., https://www.qiagenbioinformatics.com/products/ingenuity-pathway-analysis)^78^ in hard CL users (**a**) before and (**b**) after CL discontinuation. Red and green shading indicate up- and down-regulation of the proteins, respectively. Nodes (proteins) depicted with different shapes represent functional protein classes (e.g. enzymes or transmembrane receptors) and, the colours red and green represent increment and decrement of protein abundance, respectively, with different colour intensities that correspond to the degree of expression. The intensity of the node colour indicates the degree of differential regulation.

**Figure 6:**
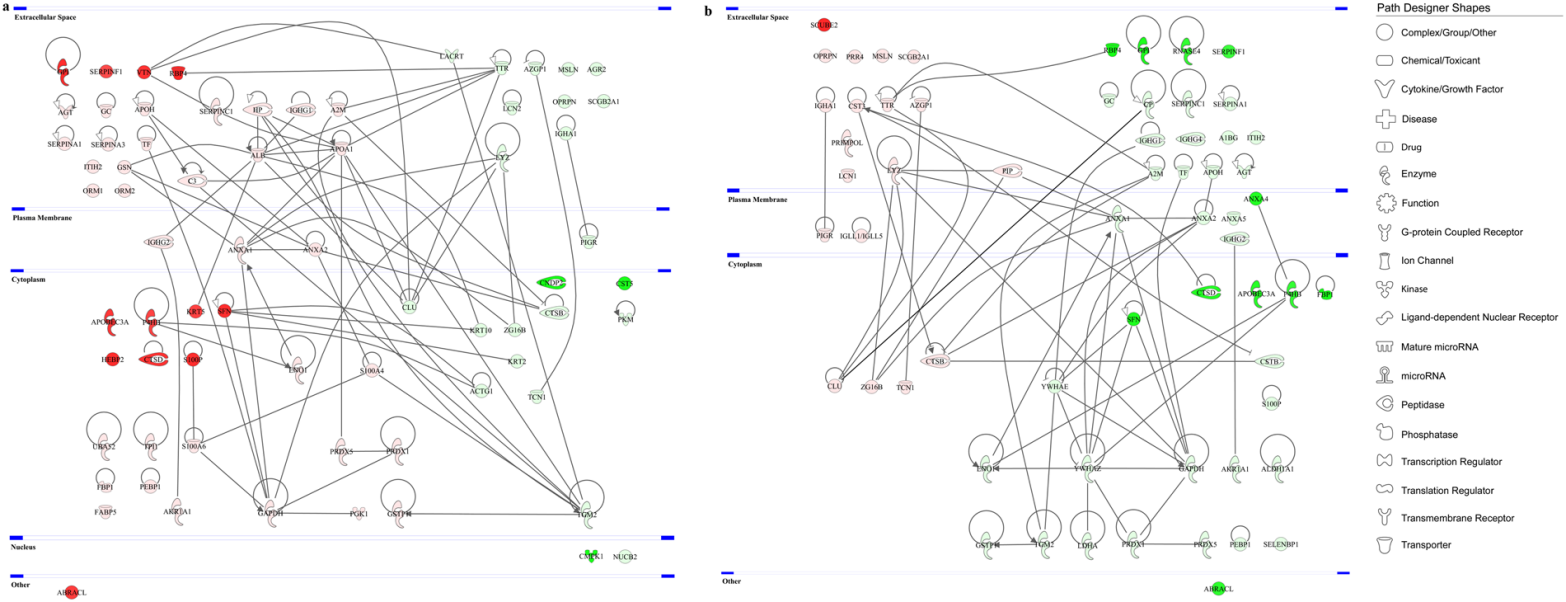
Protein-protein interaction networks of the differentially expressed tear proteins of soft CL users. The PPI networks of differentially expressed tear proteins obtained by IPA analysis (QIAGEN Inc., https://www.qiagenbioinformatics.com/products/ingenuity-pathway-analysis)^78^ in soft CL users (**a**) before and (**b**) after CL discontinuation. Red and green shading indicate up- and down-regulation of the proteins, respectively. The intensity of the node colour indicates the degree of differential regulation. The various shapes are representation of different protein functions.

Next, in an effort to further explore the protein-protein interaction (PPI) networks of the proteins identified to be differentially expressed, functional pathway enrichment was determined in each of the CL subgroups before and after discontinuation of CL use. Figures 5 and 6 depict the global view of the interactions between proteins that were differentially regulated in the tears of hard and soft CL users, respectively. A higher number of PPIs were found in the soft CL subgroups compared to its hard counterpart. In the hard CL users, the proteins with the most interactions were glyceraldehyde-3-phosphate dehydrogenase (GAPDH) and protein-glutamine gamma-glutamyltransferase 2 (TGM2) with seven PPIs each and, annexin A1 (ANXA1) and cathepsin B (CTSB) with six PPIs each (Fig. 5a). Following the renouncement of the hard CL use, apolipoprotein A-I (APOA1) was found to have the highest number of PPIs (8 PPIs), followed by GAPDH and TGM2 with six PPIs each (Fig. 5b). The use of soft CLs resulted in many proteins with high numbers of PPI, as follows: serum albumin (ALB, 12 PPIs), TGM2 (10 PPIs), GAPDH and APOA1 (8 PPIs each), and ANXA1 and transthyretin (TTR) with seven PPIs each (Fig. 6a). The renouncement of soft CLs use showed a moderately lower number of PPIs compared to its use. The top proteins with highest number of interactions involved 14-3-3 protein zeta/delta (YWHAZ, 12 PPIs), 14-3-3 protein epsilon (YWHAE) and GAPDH (8 PPIs each) and, ANXA1 with seven PPIs (Fig. 6b). In all four subgroups of CLs, GAPDH is the common protein with high PPIs.

## Discussion

It is well-documented that CL use confers several changes in the tear film proteome, especially attributed to CL-related dry eye syndrome. While there are scores of studies which have uncovered these alterations, the intricate protein interaction networks involved in the maintenance of tear film integrity in CL wearers without symptoms of dryness remain to be elucidated. This study addressed in-depth the specific changes at the protein level attributable to the use of different lens types and how these components play a vital role in an attempt to restore tear homeostasis during an acute window of renouncement of CL use. Although a recent study has demonstrated that there are no changes in the tear cytokine levels following discontinuation of CL use for 7 days^17^, our results report the contrary on the protein regulation profiles. There are several main alterations identified in the tear proteome of both hard and soft CL users attributed to the use and cessation of use of these optical devices.

One of the major proteins that was found to be exclusively downregulated in the tears of hard contact lens users is the DNA-directed primase/polymerase protein (PRIMPOL). This is a versatile enzyme that is particularly involved in ensuring efficient replication fork progression following perturbation of DNA replication and plays an important role in DNA damage tolerance^18,19^. The absence of this polymerase is most apparent in cells with challenged DNA replication, as evidenced by *PrimPol* knockdown HeLa and *PrimPol* knockout DT40 cells, which exhibited defective cell proliferation and reduction of replication fork speeds^20,21^. Ultimately, an inability to tolerate DNA damage will lead to cell death^18^. The use of hard contact lenses frequently confers mechanical damage on the cornea because they are designed to move on the eye when the wearer blinks, which could pose a significant risk of epithelial cell death and consequently lead to alterations in tear constituents^5,22,23^. Interaction of tear film with the epithelia has been shown to be pivotal in maintaining homeostasis of the ocular surface and cells at the anterior segment of the eye contribute to the tear film composition to exert protection from various insults to the eye^24^. This corresponds to the significantly high expression of 14-3-3 protein sigma (also known as stratifin, SFN) in tears of both CL users. SFN has a crucial role in governing corneal epithelial cell differentiation and promotes cell cycle arrest when DNA is damaged^25,26^. It is therefore tempting to speculate that these mechanisms at the chromosomal DNA level may represent a protective avenue to prevent further cell damage at the ocular surface attributed to the use of CLs.

Intriguingly, the expression patterns of some of the significantly differentially expressed proteins are paradoxical. One such candidate is the retinol-binding protein 4 (RBP4) which was shown to have near normal levels in the tears of hard CL but was elevated in the soft CL users. The expression of this protein, which belongs to the lipocalin family, was restored to normal levels following renouncement in the soft CL group but was increased in the hard CL post-renouncement. RBP4 is a transporter of retinol (vitamin A) and tear fluid serves as a source of this vitamin, which supplies retinol to the cornea^27^. In the blood, RBP4 forms a ternary complex with transthyretin and retinol to efficiently deliver it to target tissues as per metabolic requirement^28,29^. Retinol is an important vitamin that has essential roles in the ocular system, especially in the maintenance of the avascular cornea and conjunctival mucosa^30^. Hence, its deficiency is associated with a spectrum of ocular disorders termed as xerophthalmia^30^. Soft CLs are in contact with both conjunctiva and cornea and thus, may provoke abnormal dryness of these tissues by destabilizing the structure of the tear film, particularly within the protective lipid layer which functions to impede evaporation^31–33^. An elevation of tear osmolality as an adaptive mechanism to the use of CLs has been associated with the development of dry eye^5^. Furthermore, the augmentation of normal levels of existing regulatory molecules is often a host defence mechanism in response to antigenic stimuli and foreign objects such as CLs^12^. Since the risk of development of CL-related symptoms of dry eye is higher in soft CL wearers compared to hard CLs^34^, it can be speculated that the requirement for retinol is enhanced during the use of the former as a protective mechanism against potential development of dry eye syndrome. Once the lenses are ceased to be used, RBP4 bound to retinol returns to normal level, possibly due to the clearance activity of lipocalin since retinol is a ligand of lipocalin^35^.

On the other hand, the hard CLs are smaller in size compared to the soft CLs and therefore, have an advantage over their counterparts as they interact mechanically with only a portion of the cornea and, have higher oxygen transmissibility as well as better tear exchange^16,23,36^. The upregulation of RBP4 following renouncement may be a delayed type wound healing or cell survival mechanism. Similarly, upregulation of several other proteins was also only observed following the cessation of hard CL use, including protein S100-P (S100-P) and vitronectin (VTN). A study by Arumugam *et al*. has supported the role of S100-P as a cell growth and survival factor^37^, while VTN is well-recognized to be a highly adaptable reactive protein^38^ and its expression varies according its unique capacity to partake in divergent downstream reactions.

The VTN level in tears of soft CL users was dramatically upregulated and, this upregulation did not revert back to normal levels following renouncement. A marked elevation of this protein was also reported in tears following eyelid closure^38^ and this phenomenon corresponds with the use of soft CLs, which mimics prolonged closed-eyes state^39^. Additionally, soft CLs often cause chronic hyperaemia of the bulbar and palpebral conjunctiva and consequently, leakage of adhesive serum glycoproteins, including VTN, from dilated blood vessels into the tear film has been observed^40^. On the contrary, the levels of VTN increased only after the renouncement of hard CLs. The activation and influx of additional new components or augmentation of existing components in tears are also attributable to the frictional interaction and movement of the eyelid with the CL over the ocular surface^12^. Hard CL wearers are more exposed to fricto-mechanical stimulation than their soft counterparts owing to the material stiffness and movement of the lens edges during blinking, which could have resulted in minor injury to the cornea, however modest though that may be^5,11,23^. Although a large majority of VTN originates from conjunctiva, other main endogenous reservoirs of VTN include tear film and the basement membrane of corneal epithelium^41,42^. As such, it is actively involved in promoting corneal epithelial wound healing processes and heightens cell migration after an injury^43^. In the present circumstances, a tenable explanation for the increment of VTN observed after the discontinuation of the hard CLs could be attributed to a delayed phase protein reaction in response to post-injury corneal wound healing mechanism.

The healing processes following corneal tissue injury are not new host-defence mechanisms. However, the elucidation of the interactions between important molecular components, particularly the proteins, as a result of CL-induced injury is pivotal to better understand the mechanistic cascade that efficiently governs the subsequent healing events. A cluster of proteins associated with wound healing were found to be differentially regulated in the current study. Among the identified protein candidates, SFN was observed to be significantly upregulated following the use of both types of CLs. As per its denomination, SFN is specifically confined to the stratified layers of corneal epithelium^24,25^. Corresponding to its cellular localization, SFN plays crucial roles in differentiation of the self-renewing epithelia and most importantly, high expression of this human epithelial marker is assumed to exert protective effects on the cornea^26^. It is of interest to note that certain proteins involved in the wound healing mechanisms were only differentially expressed in the tear fluid of users of a particular CL type in this study. This is best exemplified by peroxiredoxin-6 (PRDX6), which was found to be elevated exclusively in the hard CL group and GC in the soft CLs. Although PRDX6 is widely distributed in all ocular tissues including the cornea, its physiological relevance in the eye remains to be unravelled^44^. Notwithstanding the largely unknown role of this sole mammalian 1-Cys peroxiredoxin, this protein is recognised to participate in skin wound healing^45^ and most importantly, it functions as a crucial enzyme during active corneal wound repair, with profoundly increased levels in actively proliferating epithelial cells^46^. Correspondingly, topical administration of PRDX6 on the cornea was found to maintain cellular homeostasis^44,45,47^. These assign an important role for PRDX6 in regulating cell proliferation during corneal wound healing and conferring cytoprotection against stressors^46,48^.

Although some studies do not support the premise of contact lens-induced discomfort as a result of inflammation based on the examination of tear cytokines levels^49,50^, inflammatory responses in CL users, especially in those using the soft type, can be a result of hypoxia. The use of soft CLs generally impedes the diffusion of oxygen to the ocular surface and thus, hypoxia is still one of the most prevalent factors underlying CL-associated alterations of tear homeostasis and micro-trauma on the ocular surface^1,39^. Concordantly, the results of the present investigation are in agreement with hypoxia-induced inflammation as evidenced by the regulation profiles of certain proteins that work together to ameliorate potential insults following CL use. An array of proteinases are activated and synthesized by the cornea in response to inflammation^51^ and, one of the major naturally occurring proteinase inhibitors present in the pre-corneal tears that maintains the delicate balance between protein synthesis and proteolytic degradation is alpha-2-macroglobulin (A2M)^52^. This multifunctional proteinase inhibitor with broad spectrum activity, which rapidly inhibits excessive proteinases released from cells during inflammation^53^, was found to be significantly upregulated in both CL wearers and restored to near normal levels following discontinuation of use in our investigation.

Of note, CL use also evoked a significant upregulation of glycolytic enzymes associated with hypoxia. Correspondingly, the glycolysis and gluconeogenesis pathways have been shown to be among the top canonical pathways implicated in both CLs, with a particularly significant regulation of glucose-6-phosphate isomerase (GPI). A similar phenomenon has been reported by Naughton, where hypoxia-induced increment of GPI perpetuates rheumatoid arthritis because *GPI* was identified as a hypoxic inducible gene activated *via* the hypoxia-inducible factor-1 (HIF-1) transcription factor^54–56^. In the present scenario, the regulation of GPI differs between the CL groups following renouncement because the discontinuation of use of the hard CLs did not alter the GPI level to normalcy but was restored to normal level in the soft CL group. This discrepancy is typified by fact that the movement dynamics and geometrical properties of the hard CLs differ from those of the soft lenses, and thereby, trigger germane downstream reactions. A study by Murphy *et al*. has elegantly proposed that the users of hard CLs are affected predominantly by a mechanical effect but not as significantly by metabolic effects as in soft CL wearers^23^. Mechanical abrasion of the cornea that emerges from the use of hard CLs may result in the leakage of glucose from damaged epithelial cells and diffuse directly into the tears^57,58^. Both glycolysis and gluconeogenesis pathways are pertinent for cell survival and glucose is an important metabolite for corneal wound healing^22^. Nevertheless, the use of hard CLs is known to take a longer recovery period to restore normal corneal nerve function as assessed by corneal sensitivity compared to their soft counterparts^23^, and this could be one factor that explains the levels of GPI that remained upregulated even after the lenses are discontinued to be used.

Conversely, the use of soft CLs contributes significantly to the development of hypoxia owing to a large coverage of and relatively tight fitting on the ocular surface^39^. Hypoxic cells are known to alter their cellular activities to adapt to this insult by slowing proliferation rate and enhance glycolysis^59^. Another top canonical pathway activated during the use of soft CLs in the current study is the farnesoid X receptor/retinoid X receptor (FXR/RXR) pathway. The FXR is a sub-cluster of metabolic receptors, which binds to RXR to regulate gene expressions involved in cognate metabolic pathways, including glucose metabolism, for promoting tissue regeneration and restoring homeostasis after an injury^60^. Following the removal of soft CLs, the metabolic demand of the ocular surface is restored and hence, the observed reversion of GPI to normal levels.

An interesting observation in the present study is that several proteins involved in reflex tearing, including lacrimal gland-associated proteins, were appreciably decreased in the tears of CL wearers. These mainly comprise extracellular glycoprotein lacritin (LACRT), mesothelin (MSLN) and zymogen granule protein 16 homolog B (ZG16B). In retrospect, concordant results have been reported from studies investigating the effect of CL-related dry eye, in which LACRT was among the substantially reduced proteins^5,9,61,62^. Although an impressive body of literature has accumulated about the functions of LACRT, there is still a relative dearth of information on the the physiological roles of MSLN and ZG16B in tears. Our previous study has demonstrated that these proteins were among those that were significantly increased in abundance in the regulation of reflex tears in an attempt to lubricate and protect the ocular surface^14^. Reflex tearing involves dromic stimulation of the afferent sensory corneal and conjunctival nerves that activate efferent nerves to the lacrimal glands^63^. Sensory nerves innervating the ocular surface are also actively involved in the regulation of the corneal epithelial and conjunctival cells secretion that contains tear proteins among others^64^. The presence of a CL on the ocular surface has long been known to considerably reduce corneal sensitivity as they interact with the highly innervated ocular surface^5,65,66^ and this depression in sensation could be an adaptation mechanism to the permanent mechanical stimulation elicited by the use of CLs^67^. Consequently, tear secretion is reduced in CL wearers^66^. Taken together, our current findings provide compelling evidence to suggest that the use of CLs depresses the reflex sensation on the ocular surface and thereby, prevent the neurological stimulation that is responsible for the upsurge of this cluster of proteins in tear fluid.

There are two limitations in the present study. First, pooled tear samples were employed. This criterion was deliberately chosen to minimize inter-individual variations^7,13,68^. Since this is the foremost study that provides a comprehensive outlook into the acute tear proteome changes in the event of renouncement of CL use and comparison between the two major types of commonly used CLs, it was important to obtain an overall mechanistic insight into the complex interplay between different protein clusters. Second, the effect of CL discontinuation was only studied for a short period of time compared to previous studies, which examined the effects after cessation of lens wear for several months^69,70^. This paradigm was adapted in the current study to determine whether or not the proteins in tears and on the ocular surface have inherent compensatory capability to restore breached tear homeostasis within an acute time frame. On the other hand, it was also equally crucial to demonstrate that frequent short-term discontinuation of CL use is a healthy eye-care regime, which ensures the recovery of eyes. Nevertheless, the assessment of individual samples and the effects of longer periods of renouncement await to be elucidated and merit specific investigation in our next study.

In conclusion, the findings emerging from our study underscore the keystone roles of the protein constituents present in tear fluid, which strive to maintain tear homeostasis in the presence of foreign objects on the ocular surface in the form of CLs. Importantly, this study has provided unprecedented insights into several pivotal physiological mechanisms that govern the observed phenomena on the ocular surface according to the type of CLs used. The use of soft CLs evoked a higher differential expression of proteins and the recovery of these components to near-normal levels following renouncement was also higher in this lens group compared to the hard CLs. However, it has to be highlighted here that the cessation of CL wear does not necessarily restore the expression of certain clusters of proteins to near-normal or normal levels, although several clusters were restored to normalcy following an acute phase of renouncement. Of note, some protein clusters were only expressed following discontinuation of CL use, which may represent the upsurge of proteins with specific restorative functions that attempt to heal the ocular surface post-CL use-elicited injury. Taken together, our findings highly recommend regular periods of cessation of CL use, regardless of the lens type, to enable the ocular surface to recover and replenish the proteins that were affected during CL wear. Our study has also opened the exciting possibility to further explore the significance of the identified protein clusters in individual samples on a person-to-person basis in a larger cohort. This effort echoes the recent advancement in MS-based proteomics, which focuses on the elucidation of protein patterns rather than single biomarkers with predictive clinical value in individuals^71^.

## Materials and Methods

### Study samples

A total of 28 subjects were recruited in this study. Written informed consent was obtained from all subjects prior to their inclusion in this study, and the study protocols were approved by the ethics committee of Rhineland-Palatinate, Germany. The study design and execution was in strict adherence to the tenets of the 1964 Declaration of Helsinki. All clinical evaluations and sample collection procedures were carried out at the Department of Ophthalmology of the University Medical Centre of the Johannes Gutenberg University Mainz. The exclusion criteria in all groups were subjects with systemic diseases (e.g., Sjogren’s syndrome, autoimmune diseases and diabetes), underlying ocular conditions including dry eye disease and corneal disease or scarring, post-corneal transplantation or corneal refractive surgery, corneal, conjunctival or intraocular inflammation, history of intraocular surgery with the exception of uncomplicated cataract surgery more than three months prior to this study, history of corneal transplantation and artificial tear application within 24 h before examination. Only subjects with Schirmer wetting length of ≥10 mm in five minutes were included in the sampling. All subjects were ≥18 years old and regular CL users for at least one year on a daily-basis before the first study visit and for at least six hours on the day of the first sample collection. Basal tear samples were collected with Schirmer strips from both eyes of rigid gas permeable (hard) CL users (n = 16 samples from 8 subjects), soft CL users (n = 18 samples from 9 subjects) and a control group (CTRL; n = 22 samples from 11 subjects). Topical anaesthesia (Novesine, 0.4%) was administered in both eyes of participants five minutes before tear collection. Schirmer strips were placed in the temporal part of the inferior conjunctival fornix and eyes were closed for the next five minutes. Samples were stored immediately in −80 °C until subsequent analysis. The hard and soft CL groups were each further divided into subgroups A and B, where A represented the tear samples collected after the participants had worn the lenses and B represented tear samples collected after renouncement of contact lens use for 4.7 ± 0.7 days. The soft CLs wearers used comfilcon A lenses (Cooper Vision Biofinity and Cooper Vision Ascend Evolve Toric), lotrafilcon A lenses (Air Optix Alcon), lotrafilcon B lenses (Air Optix Aqua), narafilcon A lenses (Acuvue TruEye), methafilcon A lenses (MPG&E) and nelfilcon A lenses (Ciba Vision). The hard CL wearers used the following types of lenses: Zeiss/Wöhlk A90 Advance, Hecht Bias, Hecht Bias One, Hecht Bias Mac Bo-Eq and Hecht Ascon AS 6 Advance. This study included a random distribution of different types of soft and hard contact lenses in the respective groups in order to exclude the potential effect of particular lenses on the tear proteome.

### Sample preparation and 1DE

Tear proteins were extracted from Schirmer strips with phosphate buffered saline (PBS). Briefly, each strip was soaked in 300 μl PBS for 3 hours at 4 °C while shaking to elute the tear proteins. Next, tear protein concentration was determined in each sample employing the Pierce BCA Protein Assay Kit (Thermo Scientific, Rockford, USA). The samples in each group were equally pooled to yield a total of 50 μg with three replicates. Sample pooling and equal amount of protein in each group was used to reduce inter-individual variations and normalize differences between participants. The pooled tear samples were then subjected to 1DE (50 μg per well) employing precast 4–12% Bis-Tris 10-well mini-gels (Invitrogen, Karlsruhe, Germany) with MES running buffer under reducing conditions at a constant voltage of 150 V in 4 °C for one hour. The pre-stained protein standard, SeeBlue Plus 2 (Invitrogen, Karlsruhe, Germany), was used as a molecular mass marker and the gels were stained with Colloidal Blue Staining Kit (Invitrogen, Karlsruhe, Germany), as per the manufacturer’s instructions. Protein bands were excised (10 bands per replicate), reduced and alkylated prior to in-gel trypsin digestion employing sequence grade-modified trypsin (Promega, Madison, USA), as described in detail in our previous studies^13,72^. Peptides extracted from trypsin digestion were purified with ZipTip C18 columns (Millipore, Billerica, MA, USA) according to the manufacturer’s instructions. The combined peptide eluate was concentrated to dryness in SpeedVac and dissolved in 10 μl of 0.1% trifluoroacetic acid (TFA) prior to LC-MS/MS analysis.

### Liquid chromatography (LC) - Electrospray Ionization (ESI) - MS/MS

The LC-ESI-LTQ-Orbitrap MS system is well-established in our laboratory and has been extensively optimized to minimize ion suppression effects and to improve sequence coverage, as described in detail previously^13,14,68^. The LC system was made of Rheos Allegro pump (Thermo Scientific, Rockford, USA) coupled to an HTS PAL autosampler (CTC Analytics AG, Zwingen, Switzerland). The system comprised of a 30 × 0.5 mm BioBasic C18 precolumn (Thermo Scientific, Rockford, USA) connected to a 150 × 0.5 mm BioBasic C18 column (Thermo Scientific, Rockford, USA), the C18 being the hydrophobic alkyl chains that will have reversible hydrophobic interactions with the peptides. The reverse phase aqueous solvent A consisted of LC-MS grade water with 0.1% (v/v) formic acid and the organic solvent B consisted of LC-MS grade acetonitrile with 0.1% (v/v) formic acid. The gradient had a running time of 90 minutes per gel band, as follows; 0–50 min: 10–35% B, 50–70 min: 35–55% B, 70–75 min: 55–90% B, 75–80 min: 90% B, 80–83 min: 90–10% B and 83–90 min: 10% B^13,72^. The continuum MS data were obtained on an ESI-LTQ Orbitrap XL-MS system (Thermo Scientific, Bremen, Germany). The general parameters of the instrument were set as follows: positive ion electrospray ionization mode, a spray voltage of 2.15 KV and a heated capillary temperature of 220 °C. Data was acquired in an automatic dependent mode whereby, there was automatic acquisition switching between Orbitrap-MS and LTQ MS/MS. The Orbitrap resolution was 30000 at *m/z* 400 with survey full scan MS spectra ranging from an *m/z* of 300 to 1600. Target automatic gain control (AGC) was set at 1.0 × 10^6^ ion. Internal recalibration employed polydimethlycyclosiloxane (PCM) at *m/z* 445.120025 ions in real time^73^ and the lock mass option was enabled in MS mode. Tandem data was obtained by selecting top five most intense precursor ions and subjected them for further fragmentation by collision-induced dissociation (CID). The normalized collision energy (NCE) was set to 35% with activation time of 30 ms with repeat count of 3 and dynamic exclusion duration of 600 s. The resulting fragmented ions were recorded in the LTQ.

### Label-free quantification (LFQ) analysis

The acquired continuum MS spectra were subjected to LFQ analysis employing MaxQuant computational proteomics platform version 1.5.2.8 (http://www.maxquant.org) with a built-in Andromeda search engine for peptide and protein identification and, LFQ and intensity-based absolute quantification (iBAQ) algorithm enabled^74,75^. The tandem MS spectra were searched against UniProt database (*Homo sapiens*; date: 10^th^ July 2017) employing the following standard settings: Peptide mass tolerance of ±30 ppm, fixed modifications set to carbamidomethylation of cysteine, variable modifications assigned to oxidation of methionine and acetylation of N-termini, fragment mass tolerance set to ±0.5 Da with ≥6 amino acid residues and only ‘unique plus razor peptides’ that belong to a protein were chosen, trypsin as enzyme and maximum number of missed cleavages sites set to 2. A target-decoy based false discovery rate (FDR) of 1% was used for peptide and protein identification^75^. The MaxQuant-generated output data table “proteingroups.txt” was filtered for contaminants and reverse hits prior to statistical analysis with Perseus software (version1.5.0.31) and, subsequent functional annotation and pathway analyses. The summary of MaxQuant parameters employed in the current analyses is tabulated in Supplementary Table S1.

### Bioinformatics and functional annotation and pathways analyses

In the Perseus software, the statistical analysis was done with the following parameters: First, Pearson’s correlation coefficients were analyzed employing the normalized LFQ intensity dataset to assess experimental reproducibility and the homogeneity of the designated groups. Next, a log_2_ transformation of all LFQ intensities was done so that protein down- and up-regulations with the same magnitude possess equal distances in a visual representation like scatter plot or histogram^76^. Missing values were imputated with a constant using the standard settings in Perseus. This was followed by a Student’s two-sample t-test for all the groups with p < 0.05 to identify the significantly differentially expressed proteins. Venn diagrams were generated using a web-based analysis tool named InteractiVenn^77^. Unsupervised hierarchical clustering analysis was performed according to Euclidean distance (linkage = average; preprocess with k-means). Statistica (v13, StatSoft, Tulsa, OK) was utilized for further statistical analyses and graphical presentation of the differential expression protein profiles. The gene names of these significantly (p < 0.05) differentially expressed proteins in each group were subsequently used for functional annotation and pathways analyses employing Ingenuity Pathway Analysis software (v01–04, IPA; Ingenuity QIAGEN Redwood City, CA) (https://www.qiagenbioinformatics.com/products/ingenuity-pathway-analysis)^78^. The IPA analyses elucidated the Gene Ontology cellular component (GOCC) terms, molecular types, PPI networks, and top disease functions associated with the identified differentially expressed proteins. Top canonical pathways of the differentially expressed proteins were presented with *p*-value calculated using Benjamini-Hochberg corrected Fisher’s exact test. In PPI network analysis, proteins are represented with their corresponding gene names described as nodes and the various line relationships between them described as edges. In the trimming of the network, only protein-protein interactions experimentally observed and had direct associations were allowed.

## Electronic supplementary material

Supplementary Information

## References

[1] Tariq F, Koay P (2013). The risk of contact lens wear and the avoidance of complications. IJMS.

[2] McMahon TT, Zadnik K (2000). Twenty-five years of contact lenses: the impact on the cornea and ophthalmic practice. Cornea.

[3] Cavanagh HD, Robertson DM, Petroll WM, Jester JV (2010). Forty Years in Search of the Perfect Contact Lens. Cornea.

[4] Muntz A, Subbaraman LN, Sorbara L, Jones L (2015). Tear exchange and contact lenses: A review. J Optom.

[5] Boost M, Cho P, Wang Z (2017). Disturbing the balance: effect of contact lens use on the ocular proteome and microbiome. Clin Exp Optom.

[6] Schultz CL, Kunert KS (2000). Interleukin-6 levels in tears of contact lens wearers. J. Interferon Cytokine Res..

[7] Zhao Z (2008). Proteomic analysis of protein deposits on worn daily wear silicone hydrogel contact lenses. Mol. Vis..

[8] Craig JP (2013). The TFOS International Workshop on Contact Lens Discomfort: Report of the Contact Lens Interactions With the Tear Film SubcommitteeReport on Interactions With Tear Film. Invest. Ophthalmol. Vis. Sci..

[9] Nichols JJ, Green-Church KB (2009). Mass spectrometry-based proteomic analyses in contact lens-related dry eye. Cornea.

[10] Thai LC, Tomlinson A, Doane MG (2004). Effect of contact lens materials on tear physiology. Optom Vis Sci.

[11] Willcox MD (2017). TFOS DEWS II tear film report. Ocul Surf.

[12] Mann A, Tighe B (2013). Contact lens interactions with the tear film. Exp. Eye Res..

[13] Perumal, N., Funke, S., Pfeiffer, N. & Grus, F. H. Proteomics analysis of human tears from aqueous-deficient and evaporative dry eye patients. *Sci. Rep*. **6** (2016). 10.1038/srep29629 PMC4951640 27436115

[14] Perumal N, Funke S, Wolters D, Pfeiffer N, Grus FH (2015). Characterization of human reflex tear proteome reveals high expression of lacrimal proline‐rich protein 4 (PRR4). Proteomics.

[15] Grus FH (2005). Effects of multipurpose contact lens solutions on the protein composition of the tear film. Cont Lens Anterior Eye.

[16] Kramann C (2011). Effect of contact lenses on the protein composition in tear film: a ProteinChip study. Graefes Arch. Clin. Exp. Ophthalmol..

[17] Chao C, Golebiowski B, Stapleton F, Richdale K (2016). Changes in Tear Cytokine Concentrations Following Discontinuation of Soft Contact Lenses—A Pilot Study. Eye & Contact Lens.

[18] Guilliam TA, Doherty AJ (2017). PrimPol—prime time to reprime. Genes.

[19] Rudd SG, Bianchi J, Doherty AJ (2014). PrimPol—A new polymerase on the block. Mol Cell Oncol.

[20] Bianchi J (2013). PrimPol bypasses UV photoproducts during eukaryotic chromosomal DNA replication. Mol Cell.

[21] Mourón S (2013). Repriming of DNA synthesis at stalled replication forks by human PrimPol. Nat. Struct. Mol. Biol..

[22] Karamichos D (2015). Tear metabolite changes in keratoconus. Exp. Eye Res..

[23] Murphy PJ, Patel S, Marshall J (2001). The effect of long-term, daily contact lens wear on corneal sensitivity. Cornea.

[24] Shankardas J, Senchyna M, Dimitrijevich SD (2008). Presence and distribution of 14-3-3 proteins in human ocular surface tissues. Mol. Vis..

[25] Lu Q, Xin Y, Ye F, Foulks G, Li Q (2011). 14-3-3σ controls corneal epithelium homeostasis and wound healing. Invest. Ophthalmol. Vis. Sci..

[26] Zanello SB, Nayak R, Zanello LP, Farthing-Nayak P (2006). Identification and Distribution of 14.3. 3σ (Stratifin) in the Human Cornea. Curr. Eye Res..

[27] Ubels JL, Foley K, Rismondo V (1986). Retinol secretion by the lacrimal gland. Invest. Ophthalmol. Vis. Sci..

[28] Kotnik P, Fischer-Posovszky P, Wabitsch M (2011). RBP4: a controversial adipokine. Eur. J. Endocrinol..

[29] Noa N (2000). Retinoid-binding proteins: mediators of retinoid action. Biochem. J..

[30] Smith J, Steinemann TL (2000). Vitamin A deficiency and the eye. Int Ophthalmol Clin.

[31] Efron N (2017). Contact lens wear is intrinsically inflammatory. Clin Exp Optom.

[32] Glasson M, Stapleton F, Keay L, Willcox M (2006). The effect of short term contact lens wear on the tear film and ocular surface characteristics of tolerant and intolerant wearers. Cont Lens Anterior Eye.

[33] Lorentz H, Jones L (2007). Lipid deposition on hydrogel contact lenses: how history can help us today. Optom Vis Sci.

[34] Riley C, Young G, Chalmers R (2006). Prevalence of ocular surface symptoms, signs, and uncomfortable hours of wear in contact lens wearers: the effect of refitting with daily-wear silicone hydrogel lenses (senofilcon a). Eye & Contact lens.

[35] Redl B (2000). Human tear lipocalin. BBA - Protein Structure and Molecular Enzymology.

[36] Jones LW, Jones DA (2001). Non-inflammatory corneal complications of contact lens wear. Cont Lens Anterior Eye.

[37] Arumugam T, Simeone DM, Schmidt AM, Logsdon CD (2004). S100P stimulates cell proliferation and survival via receptor for activated glycation end products (RAGE). J. Biol. Chem..

[38] Sack RA, Underwood PA, Tan KO, Sutherland H, Morris CA (1993). Vitronectin: possible contribution to the closed-eye external host-defense mechanism. Ocul. Immunol. Inflamm..

[39] Liesegang TJ (2002). Physiologic changes of the cornea with contact lens wear. Eye & Contact Lens.

[40] Baleriola-Lucas C, Fukuda M, Willcox M, Sweeney D, Holden B (1997). Fibronectin concentration in tears of contact lens wearers. Exp. Eye Res..

[41] Sack R, Underwood A, Tan K, Morris C (1994). Vitronectin in human tears–protection against closed eye induced inflammatory damage. Adv. Exp. Med. Biol..

[42] Xiao J (2005). Vitronectin: a possible determinant of adenovirus type 19 tropism for human corneal epithelium. Am J Ophthalmol.

[43] Chow S, Di Girolamo N (2014). Vitronectin: A Migration and Wound Healing Factor for Human Corneal Epithelial CellsCorneal Wound Healing With Vitronectin. Invest. Ophthalmol. Vis. Sci..

[44] Tchah H (2005). Regulation of 1-cys peroxiredoxin expression in the process of stromal wound healing after photorefractive keratectomy. Invest. Ophthalmol. Vis. Sci..

[45] Manevich Y, Fisher AB (2005). Peroxiredoxin 6, a 1-Cys peroxiredoxin, functions in antioxidant defense and lung phospholipid metabolism. Free Radic. Biol. Med..

[46] Pak JH, Choi H-j, Choi CY, Tchah H (2006). Expression of 1-cys peroxiredoxin in the corneal wound-healing process. Cornea.

[47] Shi H (2012). Topical Administration of Peroxiredoxin-6 on the Cornea Suppresses Inflammation and Neovascularization Induced by Ultraviolet RadiationPRDX6 Inhibited Inflammation and Neovascularization. Invest. Ophthalmol. Vis. Sci..

[48] Fatma N (2011). Deficiency of Prdx6 in lens epithelial cells induces ER stress response-mediated impaired homeostasis and apoptosis. Am J Physiol Cell Physiol.

[49] López–de la Rosa A (2016). Corneal sensitivity and inflammatory biomarkers in contact lens discomfort. Optom Vis Sci.

[50] Willcox M, Zhao Z, Naduvilath T (2015). Cytokine changes in tears and relationship to contact lens discomfort. Mol. Vis..

[51] Twining SS (1994). Alpha 2-macroglobulin is present in and synthesized by the cornea. Invest. Ophthalmol. Vis. Sci..

[52] Ollivier F (2007). Proteinases of the cornea and preocular tear film. Vet Ophthalmol.

[53] Rehman AA, Ahsan H, Khan FH (2013). alpha‐2‐Macroglobulin: a physiological guardian. J. Cell. Physiol.

[54] Kondoh H, Lleonart M, Bernard D, Gil J (2007). Protection from oxidative stress by enhanced glycolysis; a possible mechanism of cellular immortalization. Histol. Histopathol..

[55] Naughton D (2003). Hypoxia-induced upregulation of the glycolytic enzyme glucose-6-phosphate isomerase perpetuates rheumatoid arthritis. Med. Hypotheses.

[56] Yoon D (2001). Identification of genes differentially induced by hypoxia in pancreatic cancer cells. Biochem. Biophys. Res. Commun..

[57] Baca JT, Finegold DN, Asher SA (2007). Tear glucose analysis for the noninvasive detection and monitoring of diabetes mellitus. Ocul Surf.

[58] Farandos NM, Yetisen AK, Monteiro MJ, Lowe CR, Yun SH (2015). Contact lens sensors in ocular diagnostics. Adv Healthc Mater.

[59] Shen M (2016). Quantitative proteomic analysis of mice corneal tissues reveals angiogenesis-related proteins involved in corneal neovascularization. BBA-Proteins and Proteomics.

[60] Wang Y-D, Chen W-D, Moore DD, Huang W (2008). FXR: a metabolic regulator and cell protector. Cell Res..

[61] Green-Church KB, Nichols JJ (2008). Mass spectrometry-based proteomic analyses of contact lens deposition. Mol. Vis..

[62] Karnati R, Laurie DE, Laurie GW (2013). Lacritin and the tear proteome as natural replacement therapy for dry eye. Exp. Eye Res..

[63] Acosta MC (2004). Tear secretion induced by selective stimulation of corneal and conjunctival sensory nerve fibers. Invest. Ophthalmol. Vis. Sci..

[64] Dartt DA (2009). Neural regulation of lacrimal gland secretory processes: relevance in dry eye diseases. Prog Retin Eye Res.

[65] Cruzat A, Pavan-Langston D, Hamrah P (2010). *In Vivo* Confocal Microscopy of Corneal Nerves: Analysis and Clinical Correlation. Semin Ophthalmol.

[66] Maurya R (2014). Immunoglobulin concentration in tears of contact lens wearers. J Ophthalmic Vis Res.

[67] Oliveira‐Soto L, Efron N (2003). Morphology of corneal nerves in soft contact lens wear. A comparative study using confocal microscopy. Ophthalmic Physiol Opt.

[68] Perumal N, Funke S, Pfeiffer N, Grus F (2014). Characterization of lacrimal proline-rich protein 4 (PRR4) in human tear proteome. Proteomics.

[69] Holden BA, Sweeney DF (1991). The significance of the microcyst response: a review. Optom Vis Sci.

[70] Millodot M (1978). Effect of Long-term Wear of Hard Contact Lenses on Corneal Sensitivity. Arch Ophthalmol.

[71] Geyer P, Holdt L, Teupser D, Mann M (2017). Revisiting biomarker discovery by plasma proteomics. Mol. Syst. Biol..

[72] Manicam C, Perumal N, Pfeiffer N, Grus F, Gericke A (2016). First insight into the proteome landscape of the porcine short posterior ciliary arteries: Key signalling pathways maintaining physiologic functions. Sci. Rep..

[73] Olsen JV (2005). Parts per million mass accuracy on an Orbitrap mass spectrometer via lock mass injection into a C-trap. Mol. Cell Proteomics.

[74] Cox J (2014). Accurate proteome-wide label-free quantification by delayed normalization and maximal peptide ratio extraction, termed MaxLFQ. Mol. Cell Proteomics.

[75] Cox J, Mann M (2008). MaxQuant enables high peptide identification rates, individualized ppb-range mass accuracies and proteome-wide protein quantification. Nat. Biotechnol..

[76] Tyanova S, Temu T, Cox J (2016). The MaxQuant computational platform for mass spectrometry-based shotgun proteomics. Nat Protoc.

[77] Heberle H, Meirelles G, da Silva F, Telles G, Minghim R (2015). InteractiVenn: a web-based tool for the analysis of sets through Venn diagrams. BMC Bioinformatics.

[78] Krämer A, Green J, Pollard J, Tugendreich S (2013). Causal analysis approaches in ingenuity pathway analysis. Bioinformatics.

