# Supplementary material for: Proteomics Unravels the Regulatory Mechanisms in Human Tears Following Acute Renouncement of Contact Lens Use: A Comparison between Hard and Soft Lenses
Source: Sci Rep. 2018 Aug 1;8:11526. doi: 10.1038/s41598-018-30032-5 (PMC6070515; doi:10.1038/s41598-018-30032-5)
Supplement: Supplementary file 1 — Supplementary Information [file 41598_2018_30032_MOESM1_ESM.pdf]

SUPPLEMENTARY INFORMATION

Proteomics Unravels the Regulatory Mechanisms in Human Tears Following Acute Renouncement of Contact Lens Use: A Comparison between Hard and Soft Lenses

Caroline Manicam[§*], Natarajan Perumal[§], Joanna Wasielica-Poslednik, Yong Cajetan Ngongkole, Alexandra Tschäbunin, Marcel Sievers, Walter Lisch, Norbert Pfeiffer, Franz H. Grus and Adrian Gericke

Department of Ophthalmology, University Medical Centre of the Johannes Gutenberg University Mainz, 55131 Mainz, Germany.

[§] Both authors contributed equally to this work

***Corresponding author:**

Dr. Caroline Manicam

Department of Ophthalmology,

University Medical Centre of the Johannes Gutenberg

University Mainz,

Langenbeckstr. 1,

55131 Mainz, Germany.

E-mail: cmjc_82@yahoo.com

Tel: +49 6131 17 8276

ORCID, Caroline Manicam: 0000-0001-5891-1155

ORCID, Natarajan Perumal: 0000-0002-2612-5907

Table S1 MaxQuant Parameters

MaxQuant Parameter	Value
Version	1.5.2.8
User name	aulab-p
Machine name	WS-AU-62214
Date of writing	06/29/2017 01:39:40
Fixed modifications	Carbamidomethyl (C)
Decoy mode	revert
Special AAs	KR
Include contaminants	True
MS/MS tol. (FTMS)	20 ppm
Top MS/MS peaks per 100 Da. (FTMS)	12
MS/MS deisotoping (FTMS)	True
MS/MS tol. (ITMS)	0.5 Da
Top MS/MS peaks per 100 Da. (ITMS)	8
MS/MS deisotoping (ITMS)	False
MS/MS tol. (TOF)	40 ppm
Top MS/MS peaks per 100 Da. (TOF)	10
MS/MS deisotoping (TOF)	True
MS/MS tol. (Unknown)	0.5 Da
Top MS/MS peaks per 100 Da. (Unknown)	8
MS/MS deisotoping (Unknown)	False
PSM FDR	0.01
Protein FDR	0.01
Site FDR	0.01
Use Normalized Ratios For Occupancy	True
Min. peptide Length	6
Min. score for unmodified peptides	0
Min. score for modified peptides	40
Min. delta score for unmodified peptides	0
Min. delta score for modified peptides	6
Min. unique peptides	0
Min. razor peptides	1
Min. peptides	1
Use only unmodified peptides and	True
Modifications included in protein quantification	Acetyl (Protein N-term);Oxidation (M)
Peptides used for protein quantification	Razor
Discard unmodified counterpart peptides	True
Min. ratio count	2
Re-quantify	False
Use delta score	False
iBAQ	True
iBAQ log fit	True
Match between runs	True
Matching time window [min]	5
Alignment time window [min]	20
Find dependent peptides	False
Fasta file	Database - Uniprot; Taxonomy - Human; Date - 10 July 2017
Labeled amino acid filtering	True
Site tables	Oxidation (M)Sites.txt
Decoy mode	revert
Special AAs	KR
Include contaminants	True
RT shift	False
Advanced ratios	True
AIF correlation	0.47
First pass AIF correlation	0.8
AIF topx	20
AIF min mass	0
AIF SIL weight	4
AIF ISO weight	2
AIF iterative	True
AIF threshold FDR	0.01

Table S2 List of identified tear proteins

Protein IDs	Protein names	Gene names	Razor + unique peptides	Unique peptides	Sequence coverage [%]	Unique + razor sequence coverage [%]	Unique sequence coverage [%]	Mol. weight [kDa]	Sequence length	Score	Intensity	LFQ intensity CTRL_R1	LFQ intensity CTRL_R2	LFQ intensity CTRL_R3	LFQ intensity HARD_A_R1	LFQ intensity HARD_A_R2	LFQ intensity HARD_A_R3	LFQ intensity HARD_B_R1	LFQ intensity HARD_B_R2	LFQ intensity HARD_B_R3	LFQ intensity SOFT_A_R1	LFQ intensity SOFT_A_R2	LFQ intensity SOFT_A_R3	LFQ intensity SOFT_B_R1	LFQ intensity SOFT_B_R2	LFQ intensity SOFT_B_R3
P31946	14-3-3 protein beta/alpha	YWHAB	7	5	35.8	35.8	28.5	28.1	246	128.3	96619000	20781000	15377000	22730000	32169000	35371000	34317000	16641000	22102000	18200000	16296000	21541000	25507000	11552000	0	0
P62258	14-3-3 protein epsilon	YWHAE	5	5	32.5	25.5	25.5	29.2	255	72.0	40624000	8151800	6972500	6274700	5508500	4549300	6151700	5224200	8540300	5767000	6270400	8681200	8882200	3447000	4136700	4724300
P31947	14-3-3 protein sigma	SFN	3	3	21.4	14.1	14.1	27.8	248	12.2	14099000	0	0	0	3837200	3203900	1982800	2840800	4118100	5761900	3134700	2222200	2559200	0	0	0
P63104	14-3-3 protein zeta/delta	YWHAZ	5	5	34.3	26.9	26.9	27.7	245	147.9	139870000	25078000	23435000	28553000	38258000	26664000	21658000	33654000	37906000	29548000	24708000	29244000	29157000	12208000	20490000	16049000
P05386	60S acidic ribosomal protein P1	RPLP1	1	1	14.0	14.0	14.0	11.5	114	3.7	9508300	0	0	0	0	0	0	0	0	0	0	0	0	1190800	0	0
P52209	6-phosphogluconate dehydrogenase, decarboxylating	PGD	4	4	12.4	12.4	12.4	53.1	483	114.5	26911000	1546700	1754000	3174500	3734900	4899100	2846400	4350800	913240	4579600	2662400	904130	2217900	1399000	1007100	1915500
O95336	6-phosphogluconolactonase	PGLS	2	2	14.3	14.3	14.3	27.5	258	7.0	10756000	0	0	0	0	9326600	0	0	0	0	0	0	0	0	0	0
P68133	Actin, alpha skeletal muscle	ACTA1	1	1	28.4	4.2	4.2	42.1	377	6.0	57841000	3416400	2849100	8728900	11988000	10727000	7328400	8024700	476590	8024300	4736700	5389500	4922600	3637800	2374800	3998000
P60709	Actin, cytoplasmic 1	ACTB	1	1	59.5	4.5	4.5	41.7	375	5.5	6602200	0	0	0	0	0	0	0	0	0	0	1254700	0	0	0	0
P63261	Actin, cytoplasmic 2	ACTG1	18	1	59.5	59.5	4.5	41.8	375	323.3	1233000000	142100000	127750000	154830000	289710000	203370000	142260000	156490000	75200000	165710000	96483000	114250000	94944000	96648000	116210000	90822000
Q01518	Adenylyl cyclase-associated protein 1	CAP1	1	1	4.8	4.8	4.8	51.9	475	5.4	5840500	0	3398400	0	0	0	0	0	0	0	0	0	0	0	0	0
P14550	Alcohol dehydrogenase [NADP(+)]	AKR1A1	5	5	20.9	20.9	20.9	36.6	325	83.3	67137000	4451800	4778700	4466200	17927000	18890000	16267000	10164000	14914000	10499000	12349000	15476000	15641000	8273100	6696300	6253600
P30838	Aldehyde dehydrogenase, dimeric NADP-preferring	ALDH3A1	5	5	14.1	14.1	14.1	50.4	453	41.0	90980000	7558300	6887400	3753000	11390000	8477500	10015000	10654000	13371000	11174000	11329000	9702700	8120200	11413000	10419000	13555000
Q04828	Aldo-keto reductase family 1 member C1	AKR1C1	4	4	20.7	20.7	20.7	36.8	323	16.2	20571000	3460500	2461700	2639800	5497700	6199800	5379200	2827200	4345400	2705600	3169800	4047600	4394600	3057700	2178200	0
P02763	Alpha-1-acid glycoprotein 1	ORM1	8	5	39.3	39.3	26.9	23.5	201	139.4	514090000	52538000	57452000	50422000	65831000	58814000	46152000	50295000	14393000	41025000	62966000	59714000	63543000	54229000	48263000	64553000
P19652	Alpha-1-acid glycoprotein 2	ORM2	2	2	25.9	13.4	13.4	23.6	201	66.2	49410000	3979500	4588000	4928000	4247800	4430500	2365600	3810000	0	4179500	5394800	5384400	6281700	4036800	2224800	0
P01011	Alpha-1-antichymotrypsin	SERPINA3	9	9	27.7	27.7	27.7	47.7	423	187.4	190730000	10687000	14863000	11433000	19214000	23126000	13598000	15650000	18077000	16616000	15980000	16243000	17761000	20863000	19089000	17299000
P01009	Alpha-1-antitrypsin	SERPINA1	30	30	67.9	67.9	67.9	46.7	418	323.3	2685900000	226290000	261980000	258330000	229060000	181040000	226010000	202630000	233940000	184520000	377750000	395970000	418680000	346980000	319360000	294280000
P04217	Alpha-1B-glycoprotein	A1BG	6	6	21.6	21.6	21.6	54.3	495	98.6	94531000	1883400	2616700	3235300	1525300	1435500	903050	966000	1066100	1574500	3403400	2802500	3179400	1308800	1378100	1912200
P02765	Alpha-2-HS-glycoprotein	AHSG	8	8	35.1	35.1	35.1	39.3	367	96.3	155450000	11610000	14436000	15716000	16019000	16368000	9448400	10807000	5618400	8257300	18079000	12313000	16442000	11880000	14104000	14011000
P01023	Alpha-2-macroglobulin	A2M	35	35	34.1	34.1	34.1	163.3	1474	323.3	439740000	27521000	40225000	35912000	100120000	78821000	90033000	24402000	24509000	31154000	58849000	93934000	80150000	16916000	26288000	25098000
O43707	Alpha-actinin-4	ACTN4	10	10	15.3	15.3	15.3	104.9	911	134.9	48698000	2726300	2885800	3053000	7131200	3418200	5360500	6510900	4853300	5548300	2732800	2499100	2366100	3304500	0	0
P06733	Alpha-enolase	ENO1	23	23	62.2	62.2	62.2	47.2	434	323.3	1476700000	109270000	128730000	140960000	215060000	261550000	126500000	136180000	45359000	137950000	175000000	164560000	183800000	74737000	112480000	128510000
P01019	Angiotensinogen	AGT	6	6	19.8	19.8	19.8	53.2	485	43.7	76861000	4832200	5787000	3756500	2528700	5113000	3203500	5025700	5372700	5577300	8372100	8188400	7828200	5660400	4459000	5490800
P04083	Annexin A1	ANXA1	17	17	54.9	54.9	54.9	38.7	346	323.3	708690000	68931000	51724000	54730000	171560000	205070000	181390000	136740000	209380000	150390000	97833000	110080000	118860000	78190000	48531000	38482000
P07355	Annexin A2	ANXA2	19	19	64.0	64.0	64.0	38.6	339	323.3	472770000	38234000	35079000	35212000	103980000	130470000	108740000	77689000	102740000	74013000	86081000	90616000	103230000	55896000	34761000	31507000
P12429	Annexin A3	ANXA3	3	3	12.4	12.4	12.4	36.4	323	30.7	43076000	5061100	3965700	4641100	6014200	7222500	6832400	3352800	5006500	3171700	3869600	6485700	4885300	3842200	2914900	4603900
P09525	Annexin A4	ANXA4	2	2	10.0	10.0	10.0	35.9	319	24.4	9975200	2006900	1772200	1413200	1987500	2183800	1790100	1722300	2642700	1997700	2030800	1955200	2049300	0	0	0
P08758	Annexin A5	ANXA5	10	10	40.6	40.6	40.6	35.9	320	156.5	148170000	29190000	23520000	22538000	35618000	36549000	31456000	22387000	34623000	25521000	27715000	30930000	32499000	8753100	11463000	13017000
O95994	Anterior gradient protein 2 homolog	AGR2	4	4	30.3	30.3	30.3	20.0	175	176.1	40491000	3954900	3312200	4023800	2959900	4615800	2555600	5829100	8498000	8088000	2964300	2271700	2200000	925600	1968200	603040
P03973	Antileukoproteinase	SLPI	1	1	9.1	9.1	9.1	14.3	132	20.6	151060000	0	0	0	0	0	0	0	0	10431000	0	0	0	0	0	0
P01008	Antithrombin-III	SERPINC1	12	12	33.6	33.6	33.6	52.6	464	121.6	116710000	9300700	11972000	7154600	17804000	13641000	13834000	9573200	10358000	6521700	14182000	14840000	16269000	12137000	13362000	10832000
P02647	Apolipoprotein A-I	APOA1	11	11	43.4	43.4	43.4	30.8	267	206.1	155670000	23008000	23475000	32929000	24843000	17471000	15585000	12205000	11617000	11850000	44399000	53967000	46080000	17449000	40518000	45864000
P02652	Apolipoprotein A-II	APOA2	2	2	30.0	30.0	30.0	11.2	100	6.8	4151700	0	0	0	0	0	0	0	0	0	0	0	0	0	0	264590
P46063	ATP-dependent DNA helicase Q1	RECQL	1	1	2.3	2.3	2.3	73.5	649	3.1	37362000	0	0	0	0	0	0	0	0	0	0	0	0	0	0	9859900
P98160	Basement membrane-specific heparan sulfate proteoglycan core protein	HSPG2	43	43	14.6	14.6	14.6	468.8	4391	323.3	328840000	12762000	20187000	13598000	52834000	47045000	48403000	31710000	39286000	38511000	10070000	22687000	21220000	6699200	9783400	10406000
P02749	Beta-2-glycoprotein 1	APOH	4	4	15.9	15.9	15.9	38.3	345	30.9	51464000	4791800	5669000	5010400	4542800	4522500	3012600	4594200	7646000	4520600	7208300	7701300	7132300	5260500	4389800	4539900
P61769	Beta-2-microglobulin	B2M	4	4	40.3	40.3	40.3	13.7	119	34.1	445040000	38728000	53698000	52135000	27276000	52622000	38853000	36137000	50950000	40327000	53355000	36465000	39627000	19761000	38759000	51356000
Q562R1	Beta-actin-like protein 2	ACTBL2	1	1	12.8	4.8	4.8	42.0	376	3.0	130020000	0	0	28700000	0	0	0	0	0	0	0	0	0	0	0	0
P07384	Calpain-1 catalytic subunit	CAPN1	1	1	2.7	2.7	2.7	81.9	714	3.4	1826100	0	0	0	0	0	0	0	0	0	0	0	51093	0	0	0
P27797	Calreticulin	CALR	1	1	3.4	3.4	3.4	48.1	417	14.9	13450000	0	0	0	0	0	0	0	0	0	0	0	0	0	0	1734000
P31944	Caspase-14	CASP14	1	1	5.8	5.8	5.8	27.7	242	9.7	949390	0	0	0	0	0	0	0	0	0	0	0	116850	0	0	0
P21964	Catechol O-methyltransferase	COMT	2	2	13.7	13.7	13.7	30.0	271	5.8	4033700	0	0	0	0	0	1568100	1727300	1684300	1813500	1526700	1177000	0	0	0	0
P07858	Cathepsin B	CTSB	5	5	21.8	21.8	21.8	37.8	339	111.7	60768000	7438700	7617900	6442900	4946600	4813700	4455700	6781300	3669400	7108100	4257200	5312000	5481600	9387200	10197000	7635700
P07339	Cathepsin D	CTSD	2	2	7.5	7.5	7.5	44.6	412	28.6	12116000	0	0	0	0	0	0	0	0	2320700	1387000	1512800	2925100	0	0	0
Q8N137	Centrobin	CNTROB	1	1	1.1	1.1	1.1	101.3	903	3.3	4542100	0	0	0	0	0	0	0	0	0	2152400	0	0	0	0	0
O15078	Centrosomal protein of 290 kDa	CEP290	1	1	0.4	0.4	0.4	290.4	2479	2.9	20296000	740110	0	0	0	0	0	0	0	0	0	0	0	0	0	0
P00450	Ceruloplasmin	CP	20	20	27.0	27.0	27.0	122.2	1065	245.7	299350000	17997000	27429000	25106000	29647000	34229000	28823000	15923000	19799000	22522000	29502000	40845000	32174000	9561700	12897000	16638000
Q15782	Chitinase-3-like protein 2	CHI3L2	5	5	20.0	20.0	20.0	43.5	390	17.2	21428000	3158800	3053800	2911700	2818800	3236900	2424400	3613500	4244800	3680600	2900600	3023600	2772800	3163200	0	0
O00299	Chloride intracellular channel protein 1	CLIC1	4	4	21.2	21.2	21.2	26.9	241	52.2	35356000	6895400	6221400	6591000	5705500	7744200	6670300	5864800	7916000	7089500	5180900	8787000	6548000	5558000	5560900	5457800
P10909	Clusterin	CLU	10	10	27.4	27.4	27.4	52.5	449	175.2	424700000	58192000	54982000	53410000	33968000	41047000	54379000	51024000	53627000	46080000	38211000	39536000	32297000	53179000	46239000	52950000
P23528	Cofilin-1	CFL1	4	4	35.5	35.5	35.5	18.5	166	140.8	73395000	11365000	12912000	12754000	13924000	11205000	10232000	12684000	17522000	10912000	10234000	9938300	3656300	11823000	18502000	12598000
P0C221	Coiled-coil domain-containing protein 175	CCDC175	1	1	2.1	2.1	2.1	93.6	793	-2.0	0	0	0	0	0	0	0	0	0	0	0	0	0	0	0	0
P01024	Complement C3	C3	47	43	38.4	38.4	36.0	187.2	1663	323.3	874630000	38833000	41835000	41790000	59432000	60239000	63389000	39435000	42613000	46863000	47443000	46812000	43456000	33593000	41115000	45824000
P0C0L5	Complement C4-B	C4B	1	1	1.5	1.5	1.5	192.8	1744	5.3	1672100	0	0	0	0	0	0	0	0	0	109100	0	0	0	0	0
P00751	Complement factor B	CFB	4	4	8.0	8.0	8.0	85.5	764	17.6	32196000	0	0	1587700	2439200	2122300	3106700	1709700	1790000	2336200	5428600	1445100	2106400	1586300	1888200	2088500
P08603	Complement factor H	CFH	4	4	3.8	3.8	3.8	139.1	1231	8.0	18261000	1137900	1378900	1432500	1502300	1215700	1298400	1085400	1211200	1317500	1426200	4070200	1678600	1694400	1756600	1335300
Q9P1F3	Costars family protein ABRACL	ABRACL	2	2	35.8	35.8	35.8	9.1	81	8.3	6598200	0	0	0	0	0	0	904720	1235000	1043500	625780	834480	1162800	0	0	0
P01040	Cystatin-A	CSTA	3	3	42.9	42.9	42.9	11.0	98	15.2	35133000	0	0	0	0	0	0	2185900	2437300	0	0	2568700	1594100	4347100	3013800	3834300
P04080	Cystatin-B	CSTB	4	4	52.0	52.0	52.0	11.1	98	77.4	94131000	4556000	3116400	4778300	5701500	9589900	7213800	2350300	3769900	2389700	4324100	4634800	4009200	3166600	2823400	3237900
P01034	Cystatin-C	CST3	2	2	19.2	19.2	19.2	15.8	146	24.3	70905000	2450000	2806000	2871800	4393200	5126100	4741700	1317900	1662200	1897900	2437400	2631800	2522200	3258000	3312200	4460100
P28325	Cystatin-D	CST5	3	3	25.4	25.4	25.4	16.1	142	34.0	8813400	1457600	1583100	1441000	0	0	0	0	0	0	0	0	0	0	0	0
P01036	Cystatin-S	CST4	11	6	73.8	73.8	42.6	16.2	141	306.9	13887000000	487630000	626300000	684720000	390970000	298450000	426170000	395120000	421110000	375540000	613380000	520370000	580720000	621170000	626120000	829510000
P01037	Cystatin-SN	CST1	3	3	57.4	36.2	36.2	16.4	141	59.8	1018200000	45351000	48020000	58401000	55701000	44606000	32216000	28958000	33129000	33098000	47847000	42645000	59656000	37437000	50873000	37236000
Q96KP4	Cytosolic non-specific dipeptidase	CNDP2	3	3	9.5	9.5	9.5	52.9	475	5.6	14182000	1910800	2238400	2121400	0	0	1717900	2598500	1833400	1705400	0	0	0	0	0	0
Q9UGM3	Deleted in malignant brain tumors 1 protein	DMBT1	22	22	35.3	35.3	35.3	260.7	2413	323.3	1512100000	118080000	171110000	142300000	109640000	80351000	131270000	160030000	160670000	177470000	66113000	138200000	104810000	71550000	105440000	124720000
P31941	DNA dC->dU-editing enzyme APOBEC-3A	APOBEC3A	2	2	13.1	13.1	13.1	23.0	199	4.9	10937000	0	0	0	0	0	0	0	3487600	3709600	1622600	1432400	957100	0	0	0
Q96LW4	DNA-directed primase/polymerase protein	PRIMPOL	2	2	2.9	2.9	2.9	64.4	560	4.0	50409000	6456300	5246400	3871500	0	0	0	3514900	3639400	3592000	4718500	3916900	4645400	8391000	7609700	5707500
Q9NYC9	Dynein heavy chain 9, axonemal	DNAH9	2	2	0.4	0.4	0.4	511.9	4486	5.0	128700000	10337000	0	0	0	0	0	0	0	0	0	0	0	0	0	0
P13639	Elongation factor 2	EEF2	2	2	3.1	3.1	3.1	95.3	858	5.5	4366800	0	0	0	0	0	0	1029800	1094000	999330	0	0	0	0	0	0
P63241	Eukaryotic translation initiation factor 5A-1	EIF5A	1	1	14.9	14.9	14.9	16.8	154	7.6	3970100	0	0	0	0	0	0	0	0	0	0	0	486740	0	0	0
Q9GZZ8	Extracellular glycoprotein lacritin	LACRT	6	6	35.5	35.5	35.5	14.2	138	93.6	3202600000	1838600000	1394100000	1592400000	167620000	161550000	167750000	347860000	304390000	306950000	356780000	388290000	387180000	418350000	502120000	693900000
P15311	Ezrin	EZR	2	2	4.4	4.4	4.4	69.4	586	34.7	24939000	1148200	1071800	1291700	3186700	5056800	3358200	1429900	1192100	2447200	2178900	1320100	878880	699420	0	0
Q01469	Fatty acid-binding protein, epidermal	FABP5	5	5	47.4	47.4	47.4	15.2	135	41.9	157910000	6112500	7379800	5013100	9766200	7164000	18446000	16695000	24948000	19080000	18503000	17212000	15737000	19007000	14783000	28848000
P30043	Flavin reductase (NADPH)	BLVRB	1	1	7.3	7.3	7.3	22.1	206	10.0	2943200	0	0	0	0	0	0	0	0	0	0	0	0	0	0	317040
P09467	Fructose-1,6-bisphosphatase 1	FBP1	3	3	12.4	12.4	12.4	36.8	338	41.5	25433000	1760100	1467400	1798300	8859500	11544000	8222100	3032900	4606600	5497000	4447800	5871100	5255600	0	0	0
P04075	Fructose-bisphosphate aldolase A	ALDOA	4	4	18.4	18.4	18.4	39.4	364	66.2	38386000	4289800	4864000	2969400	3177100	4777000	4889000	4273500	2616400	3227300	3385900	5162500	4323900	4406300	5899800	5443700
P09972	Fructose-bisphosphate aldolase C	ALDOC	1	1	6.3	6.3	6.3	39.5	364	16.8	5534400	0	0	0	0	0	0	0	0	0	0	0	0	0	1830100	0
P17931	Galectin-3	LGALS3	5	5	23.2	23.2	23.2	26.2	250	48.9	125240000	26413000	16549000	24599000	21300000	21547000	27671000	18024000	28085000	19354000	21139000	26233000	27630000	21624000	17759000	17880000
Q08380	Galectin-3-binding protein	LGALS3BP	14	14	30.8	30.8	30.8	65.3	585	199.6	344790000	19151000	20844000	22014000	14919000	16011000	22636000	33483000	25628000	25107000	27404000	13987000	17520000	17165000	27421000	24459000
O75223	Gamma-glutamylcyclotransferase	GGCT	1	1	5.9	5.9	5.9	21.0	188	3.1	1873800	0	0	0	0	0	0	0	0	0	0	0	915390	0	0	0
P06396	Gelsolin	GSN	11	11	22.6	22.6	22.6	85.7	782	249.2	365440000	9182400	11334000	12309000	30239000	36524000	17316000	17336000	20050000	16686000	15858000	14440000	16922000	13534000	14442000	15341000
P06744	Glucose-6-phosphate isomerase	GPI	2	2	5.7	5.7	5.7	63.1	558	59.8	26336000	0	0	0	4198100	6908700	3070900	2768700	3275100	2836900	3143900	3153800	2886500	0	0	0
P00390	Glutathione reductase, mitochondrial	GSR	2	2	6.3	6.3	6.3	56.3	522	7.0	16271000	0	0	0	0	0	0	0	0	0	0	0	0	0	3279000	0
Q7RTV2	Glutathione S-transferase A5	GSTA5	1	1	4.1	4.1	4.1	25.7	222	3.6	5834100	0	0	0	0	0	0	0	0	0	0	0	1755800	0	0	0
P09211	Glutathione S-transferase P	GSTP1	12	12	69.0	69.0	69.0	23.4	210	323.3	955640000	168000000	162150000	166390000	294200000	284190000	262310000	261800000	239820000	223500000	222970000	213970000	209170000	113460000	187440000	122790000
P04406	Glyceraldehyde-3-phosphate dehydrogenase	GAPDH	6	6	35.5	35.5	35.5	36.1	335	134.4	221150000	15535000	14843000	13000000	49514000	50469000	45866000	29559000	42120000	31090000	22556000	28438000	31621000	13168000	18418000	19125000
P00738	Haptoglobin	HP	16	16	45.3	45.3	45.3	45.2	406	189.8	512690000	51145000	49295000	50714000	49858000	39005000	24603000	31062000	5348200	30625000	58279000	60558000	58436000	62661000	79412000	72957000
P17066	Heat shock 70 kDa protein 6	HSPA6	1	1	8.7	1.7	1.7	71.0	643	6.4	15040000	0	0	0	0	0	0	0	0	965950	0	0	0	0	0	0
P04792	Heat shock protein beta-1	HSPB1	14	14	65.9	65.9	65.9	22.8	205	155.9	695470000	86037000	93195000	115800000	232230000	199900000	145550000	137630000	149910000	113040000	84356000	81328000	90746000	84793000	122530000	119120000
Q9Y5Z4	Heme-binding protein 2	HEBP2	2	2	13.7	13.7	13.7	22.9	205	69.7	10681000	0	0	0	0	0	0	1840200	1923300	1930600	2562700	4365300	2468000	1660100	2103900	2150900
P68871	Hemoglobin subunit beta	HBB	1	1	8.8	8.8	8.8	16.0	147	3.6	1782900	674350	0	0	0	0	0	0	0	0	0	0	0	0	0	0
P02790	Hemopexin	HPX	14	14	49.8	49.8	49.8	51.7	462	317.1	1085000000	38266000	31424000	32518000	31039000	37819000	24701000	29378000	29766000	30371000	35423000	36463000	37888000	33853000	27328000	39109000
P04196	Histidine-rich glycoprotein	HRG	1	1	2.7	2.7	2.7	59.6	525	4.4	8342000	0	0	0	0	0	0	0	0	0	0	381470	0	0	0	0
Q14520	Hyaluronan-binding protein 2	HABP2	1	1	3.4	3.4	3.4	62.7	560	-2.0	2890100000	314330000	253660000	235010000	2112200000	891360000	0	186800000	176570000	142930000	50643000	217620000	123780000	405300000	340940000	256200000
P01876	Ig alpha-1 chain C region	IGHA1	19	10	66.0	66.0	42.2	37.7	353	323.3	22335000000	2263100000	2717600000	2539500000	2206600000	2839100000	2258300000	3225500000	3900900000	3581200000	1259200000	1352500000	1563100000	2651300000	2768400000	2090600000
P0DOX5	Ig gamma-1 chain C region	IGHG1	15	7	47.0	47.0	22.3	49.0	449	247.2	5945800000	449870000	529570000	511800000	705610000	782670000	541570000	523080000	583590000	478340000	827800000	841140000	829810000	691500000	615610000	617990000
P01859	Ig gamma-2 chain C region	IGHG2	8	6	54.6	39.0	31.0	35.9	326	85.0	1210400000	84895000	106660000	91374000	115220000	120350000	89865000	97634000	121770000	97707000	133290000	134370000	143540000	120930000	117230000	101260000
P01860	Ig gamma-3 chain C region	IGHG3	3	3	34.7	14.1	14.1	41.3	377	29.1	86689000	6187400	2053200	6546400	12718000	14482000	6325000	10804000	15081000	8066300	4808600	7127500	7755900	7069400	7361900	6481400
P01861	Ig gamma-4 chain C region	IGHG4	2	2	38.2	13.5	13.5	35.9	327	23.9	104050000	9631700	12190000	1800000	21570000	27974000	15765000	9017100	12370000	8499400	14191000	15209000	14081000	11376000	8566200	9393800
P0DP01	Ig heavy chain V-I region HG3		1	1	9.4	9.4	9.4	13.0	117	9.7	18156000	0	0	0	0	0	0	0	0	0	0	0	0	4587500	0	0
P23083	Ig heavy chain V-I region V35		1	1	9.4	9.4	9.4	12.8	117	6.2	14076000	0	0	0	0	0	0	0	0	0	0	0	0	2265800	0	0
P0DP08	Ig heavy chain V-II region NEWM	IGHV4-4	3	3	26.5	26.5	26.5	13.0	117	46.8	164120000	14930000	16553000	14006000	3298200	3087800	10614000	21924000	24468000	21056000	12887000	12660000	16672000	19439000	20732000	16418000
P0DP03	Ig heavy chain V-III region CAM		1	1	24.8	9.4	9.4	12.9	117	5.9	18467000	0	0	0	0	0	0	0	0	0	0	0	0	4710900	0	0
P01780	Ig heavy chain V-III region JON		2	2	26.5	17.1	17.1	12.9	117	19.5	58102000	5169100	6087800	5481500	7697100	10241000	6950000	7700900	8483300	8235000	3942100	4613700	5199300	3889900	8088200	7112000
P0DP02	Ig heavy chain V-III region KOL		1	0	24.8	6.0	0.0	13.0	117	-2.0	3430500	0	0	0	0	0	0	0	0	0	0	0	0	0	0	849980
P01834	Ig kappa chain C region	IGKC	2	2	80.4	17.8	17.8	11.8	107	26.1	2208500000	376330000	323610000	489280000	942810000	804360000	581250000	313730000	427020000	283900000	354210000	363870000	353580000	360480000	727950000	628620000
P01594	Ig kappa chain V-I region AU		2	2	34.2	34.2	34.2	12.8	117	8.9	25324000	0	0	0	0	0	0	5456800	5542000	5908700	5004700	4459400	4818600	0	0	0
P06310	Ig kappa chain V-II region RPMI 6410	IGKV A18	1	1	10.8	10.8	10.8	13.1	120	14.7	244150000	0	0	0	0	0	0	0	0	0	0	0	0	0	65271000	0
P01619	Ig kappa chain V-III region B6		2	2	21.6	21.6	21.6	12.6	116	35.8	259200000	49032000	46888000	57568000	47782000	48571000	60936000	41186000	57079000	44345000	45518000	46419000	44043000	29847000	58494000	79265000
P01624	Ig kappa chain V-III region POM		1	1	7.6	7.6	7.6	13.1	119	7.1	48587000	0	0	0	0	0	0	0	0	0	0	0	0	8479700	0	0
P04433	Ig kappa chain V-III region VG		1	1	7.8	7.8	7.8	12.6	115	16.4	50104000	0	0	0	0	0	0	0	0	0	0	0	0	0	14245000	0
P06312	Ig kappa chain V-IV region	IGKV4-1	4	4	29.8	29.8	29.8	13.4	121	36.1	84506000	15935000	12340000	19162000	25238000	28578000	20710000	18485000	17400000	15406000	12431000	13713000	14565000	13766000	20740000	21045000
P01700	Ig lambda chain V-I region HA		1	1	11.1	11.1	11.1	12.3	117	4.0	33559000	5695700	0	0	0	0	0	0	0	0	0	0	0	0	0	0
P01701	Ig lambda chain V-I region NEW		2	2	13.7	13.7	13.7	12.2	117	3.6	13829000	0	0	0	0	0	0	2976500	3344900	3030800	2380700	2588900	2926300	3634600	3514300	3593200
P80748	Ig lambda chain V-III region LOI		1	1	13.7	13.7	13.7	12.4	117	3.2	56247000	0	0	0	0	0	0	0	0	10984000	0	0	0	0	0	0
P01717	Ig lambda chain V-IV region Hil	IGLV3-10	2	2	15.7	15.7	15.7	12.4	115	5.3	9806800	0	0	0	0	0	0	2612100	2453000	2324300	1351800	1908100	1779800	0	0	0
P0DOX6	Ig mu chain C region	IGHM	8	8	21.0	21.0	21.0	63.4	576	94.5	221290000	11954000	10027000	9078200	2626900	2786900	9790100	6501000	5603900	5605300	4084700	6781400	7825100	9363600	7361300	9982200
P01591	Immunoglobulin J chain	IGJ	8	8	64.2	64.2	64.2	18.1	159	100.6	1478000000	260840000	295250000	365440000	174090000	228130000	274110000	392890000	370520000	281600000	263940000	293430000	328250000	537740000	433300000	457950000
P0DOX8	Immunoglobulin lambda-like polypeptide 5	IGLL5	7	3	44.0	44.0	17.1	22.8	216	217.7	3034600000	610220000	612970000	710180000	461190000	499410000	273560000	719370000	840970000	620030000	455060000	548910000	603290000	634880000	828610000	760010000
P19823	Inter-alpha-trypsin inhibitor heavy chain H2	ITIH2	3	3	5.4	5.4	5.4	106.5	946	10.1	11364000	616010	808480	700110	913130	591080	970360	614440	583870	618250	1814800	2032200	1202800	402830	781130	625580
Q14624	Inter-alpha-trypsin inhibitor heavy chain H4	ITIH4	1	1	1.3	1.3	1.3	103.4	930	5.8	309790	0	0	0	0	0	0	0	0	0	370010	0	0	0	0	0
Q5JU85	IQ motif and SEC7 domain-containing protein 2	IQSEC2	2	2	1.0	1.0	1.0	161.7	1478	3.5	37184000	0	0	0	0	0	0	0	0	0	0	0	0	0	0	2825000
O75874	Isocitrate dehydrogenase [NADP] cytoplasmic	IDH1	1	1	4.1	4.1	4.1	46.7	414	7.0	2119500	0	0	0	0	0	0	922380	0	0	0	0	0	0	0	0
P14923	Junction plakoglobin	JUP	2	2	3.4	3.4	3.4	81.7	745	3.0	19100000	0	0	0	0	0	0	0	0	0	0	0	0	0	0	963020
P13645	Keratin, type I cytoskeletal 10	KRT10	31	26	56.0	56.0	50.3	59.5	593	323.3	2226300000	326420000	232630000	261870000	261860000	251420000	457740000	140890000	97040000	127320000	197150000	174270000	130690000	478930000	199960000	208120000
P02533	Keratin, type I cytoskeletal 14	KRT14	7	2	19.1	14.2	5.9	51.6	472	34.8	84320000	31156000	9008900	9489000	4947500	2927300	4042800	5254600	4469200	3407800	8820100	4044800	5435500	14039000	7012900	5215100
P08779	Keratin, type I cytoskeletal 16	KRT16	1	1	16.1	3.0	3.0	51.3	473	3.5	1556600	0	1024400	0	0	0	0	0	0	0	0	0	0	0	0	0
P35527	Keratin, type I cytoskeletal 9	KRT9	20	20	52.2	51.0	51.0	62.1	623	323.3	1505700000	241030000	173970000	168630000	29797000	97491000	56868000	87884000	87372000	75811000	183630000	116160000	126900000	238850000	107510000	90141000
Q9NSB2	Keratin, type II cuticular Hb4	KRT84	1	1	3.2	2.0	2.0	64.9	600	9.3	906970	0	328220	0	0	0	0	0	0	0	0	0	0	0	0	0
P04264	Keratin, type II cytoskeletal 1	KRT1	32	21	52.8	52.8	39.3	66.0	644	323.3	4488900000	766680000	453530000	480370000	237200000	388590000	557750000	268920000	222690000	227960000	429370000	348730000	306720000	979360000	404180000	367010000
P35908	Keratin, type II cytoskeletal 2 epidermal	KRT2	18	14	43.5	40.4	33.8	65.4	639	323.3	881050000	131200000	94164000	92702000	148080000	100840000	153640000	57396000	37870000	45627000	63409000	62268000	48503000	193010000	63017000	95886000
P13647	Keratin, type II cytoskeletal 5	KRT5	2	1	10.0	3.4	1.7	62.4	590	13.4	13547000	0	0	0	0	0	0	673100	670290	532560	592440	556220	561460	4037700	856250	760450
P04259	Keratin, type II cytoskeletal 6B	KRT6B	5	0	19.3	10.8	0.0	60.1	564	35.8	32946000	5574200	2373300	2906600	4290100	3351600	2163800	2940300	3820900	2109200	2710300	2825000	3199400	3049700	2378300	2252500
P48668	Keratin, type II cytoskeletal 6C	KRT6C	1	1	20.6	3.0	3.0	60.0	564	6.7	4434500	0	1882300	0	0	0	0	0	0	0	0	0	0	0	0	0
O60938	Keratocan	KERA	2	2	2.3	2.3	2.3	40.5	352	3.0	11375000	0	2343400	0	0	0	0	0	0	0	0	0	0	0	0	0
P01042	Kininogen-1	KNG1	2	2	3.4	3.4	3.4	72.0	644	7.8	15887000	0	0	0	0	0	0	0	0	0	0	0	0	0	0	1621600
P22079	Lactoperoxidase	LPO	17	17	34.8	34.8	34.8	80.3	712	261.6	383190000	15990000	19815000	21691000	17625000	20877000	18641000	35389000	26064000	27511000	26448000	8076800	10731000	17120000	21000000	24027000
P02788	Lactotransferrin	LTF	74	70	86.9	86.9	82.7	78.2	710	323.3	2.33E+11	9626700000	10037000000	9059100000	12315000000	11669000000	9986200000	11081000000	11735000000	10800000000	7257700000	8532700000	8851300000	9506200000	8664700000	9231600000
P02750	Leucine-rich alpha-2-glycoprotein	LRG1	2	2	12.7	12.7	12.7	38.2	347	6.5	3056700	0	0	0	0	0	0	0	0	470150	0	0	0	0	0	0
P30740	Leukocyte elastase inhibitor	SERPINB1	16	16	46.7	46.7	46.7	42.7	379	323.3	434730000	43945000	45500000	35864000	47392000	64918000	59987000	47835000	30747000	32344000	40944000	58804000	48627000	48465000	60397000	50623000
P31025	Lipocalin-1	LCN1	21	21	78.4	78.4	78.4	19.3	176	323.3	96038000000	6413400000	5704400000	5039500000	3071400000	1503500000	4826300000	6210400000	6134100000	4767600000	6237700000	6964400000	6468800000	8691500000	9239400000	8449300000

P00338	L-lactate dehydrogenase A chain	LDHA	9	9	32.8	32.8	32.8	36.7	332	79.4	68097000	14963000	7965800	8449700	21575000	23633000	18461000	9886200	17914000	10535000	9012500	9900100	11827000	5831000	2202800	4791400
Q9NRZ9	Lymphoid-specific helicase	HELLS	2	2	3.2	3.2	3.2	97.1	838	3.0	1026200000	0	0	0	0	43911000	17801000	0	0	0	0	0	0	45756000	0	0
P61626	Lysozyme C	LYZ	15	15	73.0	73.0	73.0	16.5	148	242.0	1.20E+11	3995100000	5117800000	4442900000	7610700000	4754900000	3681500000	4722500000	4994000000	5013700000	3369600000	2785000000	3572000000	6077100000	5962800000	7582300000
P40925	Malate dehydrogenase, cytoplasmic	MDH1	6	6	25.7	25.7	25.7	36.4	334	15.9	26977000	4754200	3707200	3774100	6608600	7200800	6010400	4108400	5344700	4395500	4754100	4914600	5010000	2607200	5299600	5391400
O75556	Mammaglobin-B	SCGB2A1	6	6	70.5	70.5	70.5	10.9	95	219.2	5020300000	727550000	770250000	674740000	286040000	509270000	482420000	378780000	585300000	323750000	477220000	478190000	521750000	805080000	611070000	615670000
Q13421	Mesothelin	MSLN	11	11	22.5	22.5	22.5	69.0	630	115.9	179390000	45179000	39723000	48252000	23327000	28019000	19215000	21529000	26390000	22590000	19885000	19931000	23029000	24668000	28951000	28229000
P58546	Myotrophin	MTPN	1	1	14.4	14.4	14.4	12.9	118	32.0	6782400	0	0	0	0	0	0	0	0	0	0	0	0	631700	0	0
Q8NF91	Nesprin-1	SYNE1	2	2	0.2	0.2	0.2	1011.1	8797	3.7	14414000	0	0	0	0	0	0	0	0	0	0	0	806230	0	0	0
Q8WXH0	Nesprin-2	SYNE2	2	2	0.4	0.4	0.4	796.4	6885	4.8	767210000	0	0	0	0	0	0	0	0	0	0	267930000	51519000	63864000	0	0
Q15825	Neuronal acetylcholine receptor subunit alpha-6	CHRNA6	1	1	2.6	2.6	2.6	56.9	494	3.8	271880000	0	0	0	0	0	0	0	0	0	0	0	0	103510000	0	0
P80188	Neutrophil gelatinase-associated lipocalin	LCN2	9	9	55.6	55.6	55.6	22.6	198	109.7	390210000	105420000	94653000	101340000	106110000	103800000	99334000	67588000	72550000	68009000	50676000	59121000	67930000	56120000	105050000	75383000
P61970	Nuclear transport factor 2	NUTF2	1	1	19.7	19.7	19.7	14.5	127	4.5	1006200	0	0	0	0	0	0	0	0	0	0	0	189800	0	0	0
P80303	Nucleobindin-2	NUCB2	4	4	12.1	12.1	12.1	50.2	420	16.0	54127000	6602100	11368000	10102000	0	0	0	7001300	11039000	6147900	4539900	4232700	3345900	5365100	2824500	4841800
P62937	Peptidyl-prolyl cis-trans isomerase A	PPIA	4	4	26.1	26.1	26.1	18.0	165	42.2	58455000	2059300	1922200	2120500	7948700	1935100	6284600	2148100	2621200	2483200	2990300	2982300	6138000	3416700	4647600	0
P23284	Peptidyl-prolyl cis-trans isomerase B	PPIB	2	2	8.8	8.8	8.8	23.7	216	4.5	6798100	1437400	1775400	0	2805000	0	0	1402200	1495800	1343000	1416700	1450600	0	0	0	0
Q06830	Peroxiredoxin-1	PRDX1	13	12	72.9	72.9	67.3	22.1	199	94.3	440860000	54879000	55346000	68954000	117210000	93068000	103860000	100450000	94625000	83659000	76285000	78791000	102150000	69452000	62397000	56835000
P32119	Peroxiredoxin-2	PRDX2	4	4	24.2	18.7	18.7	21.9	198	24.4	14809000	0	0	3342300	6758000	5082100	3203500	4556500	4334000	4258000	2854900	2103500	3923400	2905000	1737600	1564500
P30044	Peroxiredoxin-5, mitochondrial	PRDX5	6	6	44.9	44.9	44.9	22.1	214	82.7	131750000	3186300	4635700	3001900	9184800	7499500	6130800	2497200	5237000	5412200	8522900	14102000	11087000	3854000	4345700	3847400
P30041	Peroxiredoxin-6	PRDX6	7	7	41.1	41.1	41.1	25.0	224	26.5	56218000	4220800	2804000	6853700	36813000	39433000	33217000	8595000	13310000	14128000	5296300	3820100	3712000	5024900	7050900	7250300
P30086	Phosphatidylethanolamine-binding protein 1	PEBP1	7	7	51.9	51.9	51.9	21.1	187	133.3	152420000	23096000	22243000	24806000	31495000	45239000	33114000	30445000	29219000	26910000	41049000	42947000	41016000	38098000	35761000	32101000
P00558	Phosphoglycerate kinase 1	PGK1	6	6	23.7	23.7	23.7	44.6	417	101.1	62443000	6433600	6211400	6595600	15458000	16629000	6609700	5586700	4404000	7891100	7845100	7843100	9853000	5579100	0	0
P15259	Phosphoglycerate mutase 2	PGAM2	1	1	5.5	5.5	5.5	28.8	253	3.4	18318000	0	0	0	0	0	0	0	0	0	0	0	0	0	0	2834100
P14555	Phospholipase A2, membrane associated	PLA2G2A	1	1	12.5	12.5	12.5	16.1	144	14.7	28087000	0	0	0	0	0	0	0	0	1519000	0	0	0	0	0	0
P36955	Pigment epithelium-derived factor	SERPINF1	3	3	15.8	15.8	15.8	46.3	418	12.4	1654700	0	0	0	0	0	0	0	0	190200	314810	247590	361040	0	0	0
Q8TC59	Piwi-like protein 2	PIWIL2	1	1	1.2	1.2	1.2	109.9	973	-2.0	23215000	0	0	0	0	0	0	0	0	0	0	0	0	0	2226800	0
P01833	Polymeric immunoglobulin receptor	PIGR	34	34	51.8	51.8	51.8	83.3	764	323.3	12898000000	611870000	718720000	636330000	298840000	314920000	362370000	552460000	517000000	517850000	488020000	443420000	447060000	562690000	660080000	616210000
P07737	Profilin-1	PFN1	3	3	30.0	30.0	30.0	15.1	140	34.2	114790000	4237300	3617200	4228700	5571500	3551800	7198800	5356800	5576900	5516300	5059500	2713100	4105200	3018400	4304700	3034600
Q8WUM4	Programmed cell death 6-interacting protein	PDCD6IP	2	2	3.3	3.3	3.3	96.0	868	3.2	24011000	0	0	0	0	0	0	149290	0	0	0	0	0	0	0	0
P12273	Prolactin-inducible protein	PIP	11	11	71.9	71.9	71.9	16.6	146	281.8	34651000000	2415800000	2290100000	2145500000	1756800000	1411800000	1465400000	2237100000	2181400000	1599000000	1811800000	2511400000	2095100000	3246200000	3853100000	3523000000
Q99935	Proline-rich protein 1	PROL1	6	6	30.6	30.6	30.6	27.2	248	165.3	2322100000	107000000	83931000	94053000	121680000	175780000	172000000	63271000	72797000	79806000	47326000	46892000	59149000	97740000	79117000	98896000
Q16378	Proline-rich protein 4	PRR4	4	4	32.8	32.8	32.8	15.1	134	106.0	2898700000	583770000	402610000	661110000	594900000	494110000	372240000	474190000	276920000	235280000	601140000	620760000	473230000	828990000	836510000	739380000
P07602	Prosaposin	PSAP	1	1	2.9	2.9	2.9	58.1	524	15.0	12234000	0	0	0	0	0	0	0	0	0	896960	0	0	0	0	0
Q06323	Proteasome activator complex subunit 1	PSME1	1	1	4.8	4.8	4.8	28.7	249	4.7	3580800	0	0	0	0	0	1047800	0	0	0	0	0	0	0	0	0
Q9UL46	Proteasome activator complex subunit 2	PSME2	2	2	14.6	14.6	14.6	27.4	239	13.3	3090700	0	0	0	0	0	0	0	1757800	1802500	0	0	0	0	0	0
P49721	Proteasome subunit beta type-2	PSMB2	1	1	5.5	5.5	5.5	22.8	201	3.2	1581100	0	0	0	0	0	0	0	0	0	0	0	319230	0	0	0
P07237	Protein disulfide-isomerase	P4HB	3	3	9.1	9.1	9.1	57.1	508	12.8	6243800	0	0	0	821980	4334800	850960	683230	605450	819960	543550	603010	465600	0	0	0
P30101	Protein disulfide-isomerase A3	PDIA3	2	2	6.3	6.3	6.3	56.8	505	6.1	5729000	0	0	2077200	1893800	3638400	3075800	1299000	0	0	0	0	0	0	0	0
Q99497	Protein DJ-1	PARK7	4	4	26.5	26.5	26.5	19.9	189	11.5	8110700	0	2446800	0	0	0	1897700	1583900	1476500	1491100	2210800	1878400	2268800	2248700	1793800	1925000
P31949	Protein S100-A11	S100A11	7	7	80.0	80.0	80.0	11.7	105	242.8	82168000	4539300	9250100	6931500	10733000	9639200	7916100	11747000	18464000	13204000	763100	10218000	11396000	1073700	4495600	2168000
P26447	Protein S100-A4	S100A4	6	6	42.6	42.6	42.6	11.7	101	21.6	82031000	4813700	5589800	6939700	7499500	14227000	4774500	7804600	19961000	12338000	8352200	8613600	12783000	5954300	6759600	12186000
P06703	Protein S100-A6	S100A6	5	5	73.3	73.3	73.3	10.2	90	18.8	93956000	6912300	4412000	4391600	0	12707000	10885000	14226000	19792000	11666000	11544000	8801600	9300500	10123000	9142700	3023100
P31151	Protein S100-A7	S100A7	1	1	19.8	19.8	19.8	11.5	101	5.1	1444600	0	0	0	0	0	0	0	0	0	0	0	395800	0	0	0
P05109	Protein S100-A8	S100A8	10	10	84.9	84.9	84.9	10.8	93	125.0	1558100000	125080000	177150000	147220000	160490000	250790000	280570000	160230000	306850000	183230000	204490000	160820000	187900000	127830000	157510000	180750000
P06702	Protein S100-A9	S100A9	7	7	63.2	63.2	63.2	13.2	114	218.8	1892100000	108520000	48226000	107470000	136160000	115840000	137520000	67242000	75897000	64817000	73983000	89936000	79813000	81327000	67589000	51515000
P25815	Protein S100-P	S100P	3	3	53.7	53.7	53.7	10.4	95	29.7	27665000	0	0	0	0	0	0	4705800	6816400	5006000	3508600	2151400	3597900	1464100	1396100	934540
P21980	Protein-glutamine gamma-glutamyltransferase 2	TGM2	6	6	13.8	13.8	13.8	77.3	687	56.4	106390000	4465600	4580600	6106000	8940100	15206000	12494000	4344000	5574200	5340900	2719600	2530800	3063500	2163800	2029600	2272900
P68104	Putative elongation factor 1-alpha-like 3	EEF1A1P5	7	7	23.8	23.8	23.8	50.2	462	22.3	84590000	4787500	4891900	7681600	32898000	10973000	18524000	15389000	7199700	16256000	3484800	3918400	4477600	6547600	3589200	7245500
P14618	Pyruvate kinase PKM	PKM	13	13	38.2	38.2	38.2	57.9	531	234.6	196580000	14024000	22561000	18520000	45635000	47985000	36519000	40285000	45214000	36867000	7323700	7883600	9321600	8543100	9493100	8148000
P50395	Rab GDP dissociation inhibitor beta	GDI2	5	5	18.0	18.0	18.0	50.7	445	20.3	14019000	1781800	1561400	1761900	2859600	3848500	2627300	1583700	0	1842000	0	1642900	910450	0	0	0
P31749	RAC-gamma serine/threonine-protein kinase	AKT3	1	1	3.3	3.3	3.3	55.8	479	3.0	0	0	0	0	0	0	0	0	0	0	0	0	0	0	0	0
Q9H0U4	Ras-related protein Rab-1B	RAB1B	1	1	8.0	8.0	8.0	22.2	201	3.8	8778300	0	0	0	0	0	0	0	0	1698800	0	0	0	0	0	0
P20340	Ras-related protein Rab-6B	RAB6B	1	1	5.3	5.3	5.3	23.5	208	3.9	2869200	0	0	0	2172900	0	0	0	0	0	0	0	0	0	0	0
P62834	Ras-related protein Rap-1A	RAP1A	1	1	6.5	6.5	6.5	21.0	184	5.9	856220	0	0	0	0	611820	0	0	0	0	0	0	0	0	0	0
Q15293	Reticulocalbin-1	RCN1	1	1	3.9	3.9	3.9	38.9	331	21.1	3648300	0	0	0	0	0	0	0	0	516190	0	0	0	0	0	0
P00352	Retinal dehydrogenase 1	ALDH1A1	19	19	53.3	53.3	53.3	54.9	501	240.9	461750000	40085000	44309000	41744000	86440000	91550000	74820000	62709000	69833000	60595000	42173000	45818000	46599000	22371000	19199000	22079000
P02753	Retinol-binding protein 4	RBP4	2	2	13.9	13.9	13.9	23.0	201	66.0	15510000	0	0	0	0	0	1737200	2363200	3364200	2228600	8078400	6964300	1784500	0	0	0
Q9NXL2	Rho guanine nucleotide exchange factor 38	ARHGEF38	2	2	4.9	4.9	4.9	89.1	777	3.4	555410000	0	0	80373000	184660000	0	0	0	0	0	0	0	0	0	0	0
P34096	Ribonuclease 4	RNASE4	2	2	20.4	20.4	20.4	16.8	147	12.2	22232000	880720	913420	1056500	1415500	0	641780	913760	1019200	1024800	717110	858820	716850	0	0	0
O95968	Secretoglobin family 1D member 1	SCGB1D1	4	4	32.2	32.2	32.2	9.9	90	14.8	2241400000	195260000	344540000	326510000	149150000	255830000	50011000	260580000	361880000	190250000	307620000	206100000	266340000	384130000	329230000	386400000
Q13228	Selenium-binding protein 1	SELENBP1	11	11	30.3	30.3	30.3	52.4	472	97.8	154220000	17088000	18439000	14039000	26597000	33107000	23710000	17267000	21222000	15163000	18772000	17580000	16426000	9904500	9536700	9329100
P36873	Serine/threonine-protein phosphatase PP1-gamma catalytic subunit	PPP1CC	1	1	5.3	5.3	5.3	37.0	323	6.2	1271800	0	0	0	0	0	0	0	0	0	255810	0	0	0	0	0
P02787	Serotransferrin	TF	40	38	63.6	63.6	61.5	77.1	698	323.3	5437700000	106660000	114050000	117050000	162350000	126950000	189510000	92397000	90610000	87117000	285730000	306030000	310370000	176920000	176820000	175720000
P02768	Serum albumin	ALB	66	61	82.4	82.4	78.5	69.4	609	323.3	1.09E+11	3838800000	3815100000	3579700000	3819400000	3847800000	4569700000	3720100000	4456500000	3471600000	4042900000	4014200000	4066900000	4439300000	4045800000	4390300000
Q9NQ36	Signal peptide, CUB and EGF-like domain-containing protein 2	SCUBE2	2	2	1.6	1.6	1.6	110.0	999	3.2	69448000	0	0	0	0	0	0	0	5148600	0	0	0	0	7789800	10135000	7224900
P02814	Submaxillary gland androgen-regulated protein 3B	SMR3B	1	1	39.2	39.2	39.2	8.2	79	6.1	6124000	0	0	0	0	0	0	0	0	0	0	0	512460	0	0	0
P04179	Superoxide dismutase [Mn], mitochondrial	SOD2	1	1	6.3	6.3	6.3	24.7	222	14.8	3041900	0	0	0	0	0	0	0	0	0	0	684280	0	0	0	0
Q9UMR3	T-box transcription factor TBX20	TBX20	1	1	6.7	6.7	6.7	49.2	447	3.6	600620000	0	0	0	0	0	0	0	0	0	0	0	90698000	0	0	0
P10599	Thioredoxin	TXN	5	5	42.9	42.9	42.9	11.7	105	34.5	112970000	6102600	6328700	7585300	18743000	13248000	12821000	8020200	10900000	8193900	7681800	15295000	13673000	3944100	6782000	7603600
P19971	Thymidine phosphorylase	TYMP	1	1	3.1	3.1	3.1	50.0	482	5.0	3595200	0	0	0	2080300	0	0	0	0	0	0	0	0	0	0	0
P20061	Transcobalamin-1	TCN1	8	8	22.9	22.9	22.9	48.2	433	259.1	519310000	18870000	18909000	17951000	19056000	31364000	27641000	14572000	14300000	15404000	8111200	5893200	7324300	22443000	13790000	12145000
P37802	Transgelin-2	TAGLN2	7	7	46.7	46.7	46.7	22.4	199	143.6	33555000	6892500	7364600	5392500	9380400	6722800	4224900	8295700	7403200	9305400	7443600	3344000	1651700	3477000	3305700	4433900
P55072	Transitional endoplasmic reticulum ATPase	VCP	2	2	4.2	4.2	4.2	89.3	806	3.9	1804000	0	0	0	0	0	0	268050	214230	0	0	0	0	0	0	0
Q66K66	Transmembrane protein 198	TMEM198	1	1	2.5	2.5	2.5	39.5	360	-2.0	68992000	0	0	0	0	0	0	0	0	0	0	0	0	5425700	0	0
P02766	Transthyretin	TTR	4	4	39.5	39.5	39.5	15.9	147	70.6	287530000	12353000	15444000	16606000	11162000	12457000	10952000	17970000	8076100	18635000	9055600	9782300	10337000	18114000	14403000	18041000
P60174	Triosephosphate isomerase	TPI1	10	10	54.2	54.2	54.2	30.8	286	100.7	222290000	30645000	31533000	40462000	58224000	52285000	41267000	28471000	34224000	28143000	42410000	44803000	47764000	20715000	66516000	46376000
P67936	Tropomyosin alpha-4 chain	TPM4	1	1	3.5	3.5	3.5	33.0	284	3.2	6682700	0	0	0	0	0	0	0	0	0	0	0	0	0	0	810440
Q9BQE3	Tubulin alpha-1C chain	TUBA1C	2	2	5.1	5.1	5.1	49.9	449	4.3	1703400	0	0	0	612460	0	0	0	0	0	0	0	0	0	0	0
P07437	Tubulin beta chain	TUBB	2	2	9.9	9.9	9.9	49.7	444	16.8	5003700	0	0	0	0	1120900	844160	1445000	1964700	0	0	0	0	0	0	0
P62987	Ubiquitin-60S ribosomal protein L40	UBA52	3	3	26.6	26.6	26.6	14.7	128	99.8	152360000	9138300	10711000	10067000	19291000	28052000	9247400	16908000	30551000	20263000	23463000	15686000	16954000	19522000	15684000	17923000
P22314	Ubiquitin-like modifier-activating enzyme 1	UBA1	2	2	3.1	3.1	3.1	117.9	1058	11.7	5784500	0	0	0	0	0	1893500	833140	816460	949710	0	0	0	0	0	0
P30085	UMP-CMP kinase	CMPK1	3	3	18.4	18.4	18.4	22.2	196	44.2	6211300	2222500	2402500	2098400	3532800	4924100	2519200	2107900	0	0	0	0	0	0	0	0
Q6PIF6	Unconventional myosin-VIIb	MYO7B	2	2	0.9	0.9	0.9	241.6	2116	3.2	665550000	0	145590000	0	0	0	0	0	0	0	0	0	0	0	0	0
P18206	Vinculin	VCL	1	1	1.9	1.9	1.9	123.8	1134	3.3	665270	0	0	0	0	0	0	0	0	0	0	0	24782	0	0	0
P02774	Vitamin D-binding protein	GC	18	18	47.7	47.7	47.7	53.0	474	323.3	413530000	30658000	39399000	29478000	31975000	42601000	35255000	35555000	38184000	32163000	61585000	59965000	63540000	36939000	45286000	33186000
P04004	Vitronectin	VTN	2	2	6.3	6.3	6.3	54.3	478	15.0	16557000	0	0	0	0	0	0	1422500	2791200	1671200	1542900	1771600	1969700	1698300	1686900	1019700
Q9Y4E6	WD repeat-containing protein 7	WDR7	2	2	1.4	1.4	1.4	163.8	1490	3.0	113590000	0	0	0	0	0	0	0	0	0	0	0	4887800	0	0	0
P25311	Zinc-alpha-2-glycoprotein	AZGP1	22	22	58.4	58.4	58.4	34.3	298	323.3	12870000000	1696700000	1692300000	2002500000	1303900000	679150000	955430000	1724300000	308020000	1519900000	1146500000	1246000000	1158900000	1724000000	2173100000	1653900000
Q96DA0	Zymogen granule protein 16 homolog B	ZG16B	8	8	49.0	49.0	49.0	22.7	208	279.0	1031900000	275970000	221880000	291540000	163510000	176010000	163220000	203700000	310710000	232740000	121730000	141440000	159130000	177130000	188380000	193640000

Table S3 The degree of variances in the tear proteome between the (a) replicates and (b) groups assessed by Pearson's correlation analysis.

(a)

**Name**	**LFQ intensity CTRL_R1**	**LFQ intensity CTRL_R2**	**LFQ intensity CTRL_R3**	**LFQ intensity Hard A_R1**	**LFQ intensity Hard A_R2**	**LFQ intensity Hard A_R3**	**LFQ intensity Hard B_R1**	**LFQ intensity Hard B_R2**	**LFQ intensity Hard B_R3**	**LFQ intensity Soft A_R1**	**LFQ intensity Soft A_R2**	**LFQ intensity Soft A_R3**	**LFQ intensity Soft B_R1**	**LFQ intensity Soft B_R2**	**LFQ intensity Soft B_R3**
**LFQ intensity CTRL_R1**	**NaN**	**0.99**	**0.99**	0.92	0.90	0.98	0.99	0.98	0.97	0.98	0.98	0.99	0.98	0.97	0.97
**LFQ intensity CTRL_R2**	**0.99**	**NaN**	**1.00**	0.95	0.93	0.98	0.99	0.99	0.99	0.97	0.97	0.98	0.98	0.96	0.97
**LFQ intensity CTRL_R3**	**0.99**	**1.00**	**NaN**	0.94	0.93	0.98	0.99	0.98	0.98	0.97	0.96	0.98	0.97	0.96	0.96
**LFQ intensity Hard A_R1**	0.92	0.95	0.94	**NaN**	**0.98**	**0.95**	0.95	0.95	0.96	0.89	0.88	0.91	0.90	0.87	0.90
**LFQ intensity Hard A_R2**	0.90	0.93	0.93	**0.98**	**NaN**	**0.95**	0.94	0.95	0.96	0.86	0.86	0.89	0.86	0.82	0.85
**LFQ intensity Hard A_R3**	0.98	0.98	0.98	**0.95**	**0.95**	**NaN**	0.99	0.99	0.98	0.97	0.97	0.98	0.95	0.93	0.94
**LFQ intensity Hard B_R1**	0.99	0.99	0.99	0.95	0.94	0.99	**NaN**	**0.99**	**0.99**	0.97	0.97	0.98	0.97	0.96	0.96
**LFQ intensity Hard B_R2**	0.98	0.99	0.98	0.95	0.95	0.99	**0.99**	**NaN**	**0.99**	0.96	0.96	0.98	0.96	0.94	0.95
**LFQ intensity Hard B_R3**	0.97	0.99	0.98	0.96	0.96	0.98	**0.99**	**0.99**	**NaN**	0.95	0.94	0.96	0.95	0.93	0.94
**LFQ intensity Soft A_R1**	0.98	0.97	0.97	0.89	0.86	0.97	0.97	0.96	0.95	**NaN**	**0.99**	**1.00**	0.99	0.98	0.97
**LFQ intensity Soft A_R2**	0.98	0.97	0.96	0.88	0.86	0.97	0.97	0.96	0.94	**0.99**	**NaN**	**1.00**	0.98	0.97	0.96
**LFQ intensity Soft A_R3**	0.99	0.98	0.98	0.91	0.89	0.98	0.98	0.98	0.96	**1.00**	**1.00**	**NaN**	0.98	0.97	0.97
**LFQ intensity Soft B_R1**	0.98	0.98	0.97	0.90	0.86	0.95	0.97	0.96	0.95	0.99	0.98	0.98	**NaN**	**0.99**	**0.99**
**LFQ intensity Soft B_R2**	0.97	0.96	0.96	0.87	0.82	0.93	0.96	0.94	0.93	0.98	0.97	0.97	**0.99**	**NaN**	**0.99**
**LFQ intensity Soft B_R3**	0.97	0.97	0.96	0.90	0.85	0.94	0.96	0.95	0.94	0.97	0.96	0.97	**0.99**	**0.99**	**NaN**

(b)

	**LFQ intensity CTRL**	**LFQ intensity Hard A**	**LFQ intensity Hard B**	**LFQ intensity Soft A**	**LFQ intensity Soft B**
**LFQ intensity CTRL**	**0.995 ± 0.003**	0.946 ± 0.028	0.984 ± 0.007	0.976 ± 0.008	0.968 ± 0.007
**LFQ intensity Hard A**	0.946 ± 0.028	**0.959 ± 0.017**	0.963 ± 0.020	0.911 ± 0.048	0.890 ± 0.046
**LFQ intensity Hard B**	0.984 ± 0.007	0.963 ± 0.020	**0.993 ± 0.001**	0.964 ± 0.014	0.950 ± 0.013
**LFQ intensity Soft A**	0.976 ± 0.008	0.911 ± 0.048	0.964 ± 0.014	**0.995 ± 0.001**	0.975 ± 0.009
**LFQ intensity Soft B**	0.968 ± 0.007	0.890 ± 0.046	0.950 ± 0.013	0.975 ± 0.009	**0.993 ± 0.002**

Table S4 Differentially expressed tear proteins restored to normal or near normal levels following renouncement of hard contact lens

			Hard A/CTRL	Hard A/CTRL	Hard B/Hard A	Hard B/Hard A
**Protein ID**	**Protein name**	**Gene name**	**t-test p value**	**t-test difference**	**t-test p value**	**t-test difference**
P80303	Nucleobindin-2	NUCB2	6.74E-08	-23.1204	9.37E-08	22.8957
Q96LW4	DNA-directed primase/polymerase protein	PRIMPOL	5.13E-08	-22.2765	1.23E-12	21.7722
Q9GZZ8	Extracellular glycoprotein lacritin	LACRT	9.64E-06	-3.27048	0.000127961	0.946314
P0DP08	Ig heavy chain V-II region NEWM	IGHV4-4	0.0459192	-1.66699	0.0184549	2.23576
P01833	Polymeric immunoglobulin receptor	PIGR	0.000735725	-1.0122	0.00135836	0.705582
Q96DA0	Zymogen granule protein 16 homolog B	ZG16B	0.00691179	-0.642043	0.0400133	0.549627
P02787	Serotransferrin	TF	0.0485373	0.485277	0.00901612	-0.806985
P12429	Annexin A3	ANXA3	0.01255	0.557257	0.0203503	-0.826279
P60174	Triosephosphate isomerase	TPI1	0.0445722	0.561307	0.0133209	-0.731908
P01024	Complement C3	C3	0.000216808	0.58034	0.00276314	-0.508984
P06312	Ig kappa chain V-IV region	IGKV4-1	0.0441418	0.662347	0.0271293	-0.530631
P01008	Antithrombin-III	SERPINC1	0.049337	0.692117	0.0302766	-0.792411
P40925	Malate dehydrogenase, cytoplasmic	MDH1	0.00705303	0.701432	0.0186406	-0.522416
Q99935	Proline-rich protein 1	PROL1	0.0238565	0.707614	0.00497106	-1.10771
Q13228	Selenium-binding protein 1	SELENBP1	0.0152762	0.746237	0.0333095	-0.636588

P31946	14-3-3 protein beta/alpha	YWHAB	0.00991322	0.80884	0.0026455	-0.848101
P01034	Cystatin-C	CST3	0.00107646	0.811762	0.000723647	-1.56093
P04080	Cystatin-B	CSTB	0.044042	0.84649	0.010676	-1.40647
P01834	Ig kappa chain C region	IGKC	0.0233074	0.962351	0.012219	-1.17825
P00352	Retinal dehydrogenase 1	ALDH1A1	0.000486025	0.999151	0.0221524	-0.385969
Q04828	Aldo-keto reductase family 1 member C1	AKR1C1	0.00344507	1.00913	0.0224532	-0.82121
P10599	Thioredoxin	TXN	0.00460263	1.14729	0.0337251	-0.717325
P21980	Protein-glutamine gamma-glutamyltransferase 2	TGM2	0.00923312	1.25514	0.00771404	-1.23838
P01023	Alpha-2-macroglobulin	A2M	0.00189417	1.38653	0.00033579	-1.75099
P15311	Ezrin	EZR	0.00165127	1.69643	0.0305612	-1.23247
P98160	Basement membrane-specific heparan sulfate proteoglycan core protein	HSPG2	0.00133399	1.70064	0.0162177	-0.442127
P04406	Glyceraldehyde-3-phosphate dehydrogenase	GAPDH	3.73E-05	1.75228	0.0340754	-0.522034
P14550	Alcohol dehydrogenase [NADP(+)]	AKR1A1	1.07E-05	1.95248	0.0336889	-0.597106
P09467	Fructose-1,6-bisphosphatase 1	FBP1	0.000140307	2.50008	0.017434	-1.15092
P30041	Peroxiredoxin-6	PRDX6	0.00126659	3.07181	0.00233273	-1.63297

Table S5 Differentially expressed tear proteins that were restored to normal or near normal levels following renouncement of soft contact lenses

			**Soft A/CTRL**	**Soft A/CTRL**	**Soft B/Soft A**	**Soft B/Soft A**
**Protein ID**	**Protein name**	**Gene name**	**t-test p value**	**t-test difference**	**t-test p value**	**t-test difference**
P20061	Transcobalamin-1	TCN1	0.000536241	-1.39779	0.0194972	1.14146
Q13421	Mesothelin	MSLN	0.000560973	-1.08202	0.019067	0.381095
Q96DA0	Zymogen granule protein 16 homolog B	ZG16B	0.00537381	-0.901301	0.025062	0.412584
Q99935	Proline-rich protein 1	PROL1	0.00387322	-0.895296	0.00500459	0.847507
P01876	Ig alpha-1 chain C region	IGHA1	0.00207708	-0.850877	0.00566909	0.842374
P10909	Clusterin	CLU	0.00344228	-0.602778	0.0134948	0.472013
P25311	Zinc-alpha-2-glycoprotein	AZGP1	0.00251442	-0.59874	0.00775825	0.634696
P02766	Transthyretin	TTR	0.0129762	-0.596889	0.00301547	0.78727
O75556	Mammaglobin-B	SCGB2A1	0.00134163	-0.555702	0.0309456	0.449009
P07858	Cathepsin B	CTSB	0.0194062	-0.519465	0.00723964	0.853257
P01833	Polymeric immunoglobulin receptor	PIGR	0.00349671	-0.510782	0.0066351	0.414106
P61626	Lysozyme C	LYZ	0.0329406	-0.479423	0.00286996	1.01167

P09211	Glutathione S-transferase P	GSTP1	0.000255522	0.379524	0.0465344	-0.644709
P06733	Alpha-enolase	ENO1	0.0156537	0.472191	0.0332919	-0.764221
P02749	Beta-2-glycoprotein 1	APOH	0.00308197	0.513513	0.00185552	-0.639037
Q06830	Peroxiredoxin-1	PRDX1	0.0392907	0.517227	0.0492212	-0.439258
P01859	Ig gamma-2 chain C region	IGHG2	0.00606619	0.545203	0.0310358	-0.280219
P01009	Alpha-1-antitrypsin	SERPINA1	0.00105742	0.677276	0.0178656	-0.313815
P01008	Antithrombin-III	SERPINC1	0.0343189	0.70122	0.0379374	-0.320943
P01857	Ig gamma-1 chain C region	IGHG1	0.000484682	0.748166	0.00241463	-0.378359
P01019	Angiotensinogen	AGT	0.0127924	0.784323	0.00433753	-0.651071
P30086	Phosphatidylethanolamine-binding protein 1	PEBP1	8.21E-05	0.834803	0.0326834	-0.24179
P04083	Annexin A1	ANXA1	0.00388359	0.904558	0.0286612	-1.04397
P02774	Vitamin D-binding protein	GC	0.00243967	0.906768	0.00659062	-0.693193
P04406	Glyceraldehyde-3-phosphate dehydrogenase	GAPDH	0.00488883	0.919466	0.0337512	-0.709533

P01023	Alpha-2-macroglobulin	A2M	0.0105935	1.15943	0.00331981	-1.77033
P19823	Inter-alpha-trypsin inhibitor heavy chain H2	ITIH2	0.00882564	1.2231	0.0145121	-1.49804
P07355	Annexin A2	ANXA2	0.000100754	1.36391	0.00972864	-1.23912
P02787	Serotransferrin	TF	1.28E-05	1.4175	3.18E-05	-0.767886
P09467	Fructose-1,6-bisphosphatase 1	FBP1	0.000398519	1.62834	4.46E-09	-22.2984
P30044	Peroxiredoxin-5, mitochondrial	PRDX5	0.00469036	1.63648	0.00259444	-1.45671
P14550	Alcohol dehydrogenase [NADP(+)]	AKR1A1	0.000139363	1.6585	0.00325967	-1.03635
P36955	Pigment epithelium-derived factor	SERPINF1	3.49E-08	18.2145	3.49E-08	-18.2145
P07237	Protein disulfide-isomerase	P4HB	6.32E-09	19.0275	6.32E-09	-19.0275
Q9P1F3	Costars family protein ABRACL	ABRACL	1.77E-07	19.6917	1.77E-07	-19.6917
P31941	DNA dC->dU-editing enzyme APOBEC-3A	APOBEC3A	9.82E-08	20.3161	9.82E-08	-20.3161
P01717	Ig lambda chain V-IV region Hil	IGLV3-10	1.75E-08	20.6645	1.75E-08	-20.6645
P07339	Cathepsin D	CTSD	4.27E-07	20.8041	4.27E-07	-20.8041

P31947	14-3-3 protein sigma	SFN	1.25E-08	21.3169	1.25E-08	-21.3169
P25815	Protein S100-P	S100P	9.51E-08	21.5193	0.0157788	-1.27653
P06744	Glucose-6-phosphate isomerase	GPI	8.55E-11	21.5446	8.55E-11	-21.5446
P02753	Retinol-binding protein 4	RBP4	5.72E-06	22.1481	5.72E-06	-22.1481
P01594	Ig kappa chain V-I region AU		1.43E-10	22.1812	1.43E-10	-22.1812

Table S6 Differentially expressed tear proteins that were not restored to normal or near normal levels following renouncement of hard contact lenses

			Hard A/CTRL	Hard A/CTRL	Hard B/Hard A	Hard B/Hard A
**Protein ID**	**Protein name**	**Gene name**	**t-test p value**	**t-test difference**	**t-test p value**	**t-test difference**
P28325	Cystatin-D	CST5	1.13E-10	-20.5094	1	0
P02533	Keratin, type I cytoskeletal 14	KRT14	0.0424658	-1.83581	0.627139	0.150271
P35527	Keratin, type I cytoskeletal 9	KRT9	0.0256558	-1.80655	0.291764	0.605708
P04217	Alpha-1B-glycoprotein	A1BG	0.0381718	-1.00382	0.781421	-0.0953973
Q13421	Mesothelin	MSLN	0.00636721	-0.928525	0.95663	0.0104383
P25311	Zinc-alpha-2-glycoprotein	AZGP1	0.0313137	-0.921553	0.979942	-0.0225919
O75556	Mammaglobin-B	SCGB2A1	0.0405104	-0.809256	0.979318	0.0101598
P01036	Cystatin-S	CST4	0.0316764	-0.690727	0.535037	0.109827
P07858	Cathepsin B	CTSB	0.00250325	-0.594305	0.473023	0.245778
P12273	Prolactin-inducible protein	PIP	0.00647886	-0.569149	0.116147	0.367425
P08758	Annexin A5	ANXA5	0.024728	0.468018	0.151901	-0.349899
P01780	Ig heavy chain V-III region JON		0.0373057	0.555737	0.962035	-0.00873057
P30086	Phosphatidylethanolamine-binding protein 1	PEBP1	0.0207136	0.629482	0.13004	-0.326293

P09211	Glutathione S-transferase P	GSTP1	0.000122387	0.758188	0.0592956	-0.214745
Q06830	Peroxiredoxin-1	PRDX1	0.00492382	0.811836	0.240345	-0.170243
P50395	Rab GDP dissociation inhibitor beta	GDI2	0.00864002	0.85346	0.324602	-7.7469
P04792	Heat shock protein beta-1	HSPB1	0.0156339	0.954453	0.0925576	-0.511541
P00338	L-lactate dehydrogenase A chain	LDHA	0.0251644	1.07479	0.0555952	-0.778293
P30044	Peroxiredoxin-5, mitochondrial	PRDX5	0.013781	1.08386	0.0990585	-0.85895
P14618	Pyruvate kinase PKM	PKM	0.00570498	1.25685	0.600758	-0.0840111
P06396	Gelsolin	GSN	0.0199829	1.30002	0.160116	-0.573783
P07355	Annexin A2	ANXA2	0.000105848	1.65505	0.0690037	-0.440093
P04083	Annexin A1	ANXA1	0.000343761	1.67713	0.400233	-0.189219
P68104	Putative elongation factor 1-alpha-like 3	EEF1A1P5	0.026819	1.73869	0.34821	-0.630824
P07237	Protein disulfide-isomerase	P4HB	1.33E-05	20.465	0.259079	-1.05339
P31947	14-3-3 protein sigma	SFN	1.84E-07	21.4674	0.298178	0.489133
P06744	Glucose-6-phosphate isomerase	GPI	3.39E-07	22.0905	0.162099	-0.597249

Table S7 Differentially expressed tear proteins that were not restored to normal or near normal levels following renouncement of soft contact lenses

			**Soft A/CTRL**	**Soft A/CTRL**	**Soft B/Soft A**	**Soft B/Soft A**
**Protein ID**	**Protein name**	**Gene name**	**t-test p value**	**t-test difference**	**t-test p value**	**t-test difference**
P30085	UMP-CMP kinase	CMPK1	3.10E-10	-21.0936	1	0
Q96KP4	Cytosolic non-specific dipeptidase	CNDP2	6.24E-10	-20.9921	1	0
P28325	Cystatin-D	CST5	1.13E-10	-20.5094	1	0
Q9GZZ8	Extracellular glycoprotein lacritin	LACRT	6.91E-05	-2.08325	0.0912392	0.480766
P80303	Nucleobindin-2	NUCB2	0.0121563	-1.18659	0.850738	0.0635026
P14618	Pyruvate kinase PKM	PKM	0.00686079	-1.1482	0.463375	0.0986938
P35908	Keratin, type II cytoskeletal 2 epidermal	KRT2	0.0138746	-0.860073	0.149141	0.868804
P80188	Neutrophil gelatinase-associated lipocalin	LCN2	0.00408174	-0.770945	0.262706	0.375579
CON__P13645	Keratin, type I cytoskeletal 10	KRT10	0.0342843	-0.715617	0.183926	0.716726
O95994	Anterior gradient protein 2 homolog	AGR2	0.0201302	-0.610341	0.0728174	-1.2511

P63261	Actin, cytoplasmic 2	ACTG1	0.0154208	-0.475075	0.932412	-0.0123431
P02768	Serum albumin	ALB	0.0275545	0.110739	0.114849	0.0855484
P01024	Complement C3	C3	0.0332818	0.16916	0.212821	-0.2029
P02763	Alpha-1-acid glycoprotein 1	ORM1	0.0252244	0.216868	0.253601	-0.166636
P00738	Haptoglobin	HP	0.000664556	0.229911	0.055372	0.27194
P19652	Alpha-1-acid glycoprotein 2	ORM2	0.043632	0.340018	0.322546	-8.09206
P00558	Phosphoglycerate kinase 1	PGK1	0.0235661	0.400577	0.105992	-15.5423
P60174	Triosephosphate isomerase	TPI1	0.0413993	0.404948	0.75273	-0.168723
P01011	Alpha-1-antichymotrypsin	SERPINA3	0.042634	0.448003	0.101813	0.193193
P06396	Gelsolin	GSN	0.0201163	0.532289	0.21816	-0.123309
P26447	Protein S100-A4	S100A4	0.0374457	0.766703	0.465031	-0.302301
P02647	Apolipoprotein A-I	APOA1	0.00963617	0.878042	0.257336	-0.589225
P62987	Ubiquitin-60S ribosomal protein L40	UBA52	0.00950648	0.887589	0.77315	-0.0617619
P06703	Protein S100-A6	S100A6	0.0192174	0.9396	0.363222	-0.58531

Q01469	Fatty acid-binding protein, epidermal	FABP5	0.00103605	1.49002	0.468924	0.231202
P13647	Keratin, type II cytoskeletal 5	KRT5	2.90E-11	19.1201	0.175568	1.27628
P04004	Vitronectin	VTN	3.51E-09	20.7411	0.328128	-0.294021
Q9Y5Z4	Heme-binding protein 2	HEBP2	1.39E-07	21.5273	0.0981464	-0.625937

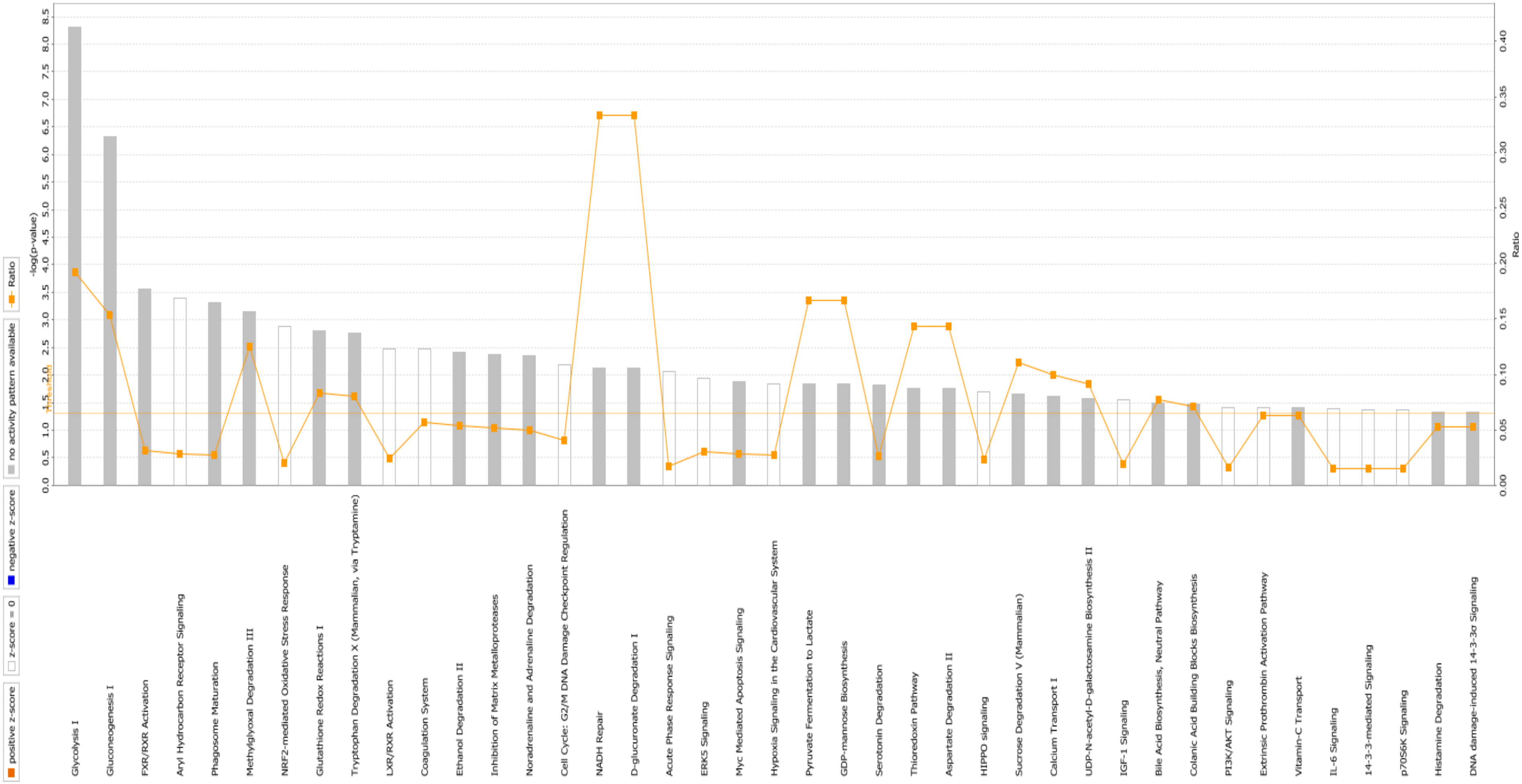

Figure S1. Canonical pathways of hard CL tear proteins. Top canonical pathways found to be significantly ($p < 0.05$) altered following the use of hard CLs identified by Ingenuity Pathway Analysis. The p-values of the proteins within the each canonical pathway was determined using the Benjamini-Hochberg corrected Fisher's exact test and ratio indicate the number of molecules in the dataset associated with a pathway to the total number of molecules identified in that particular pathway.

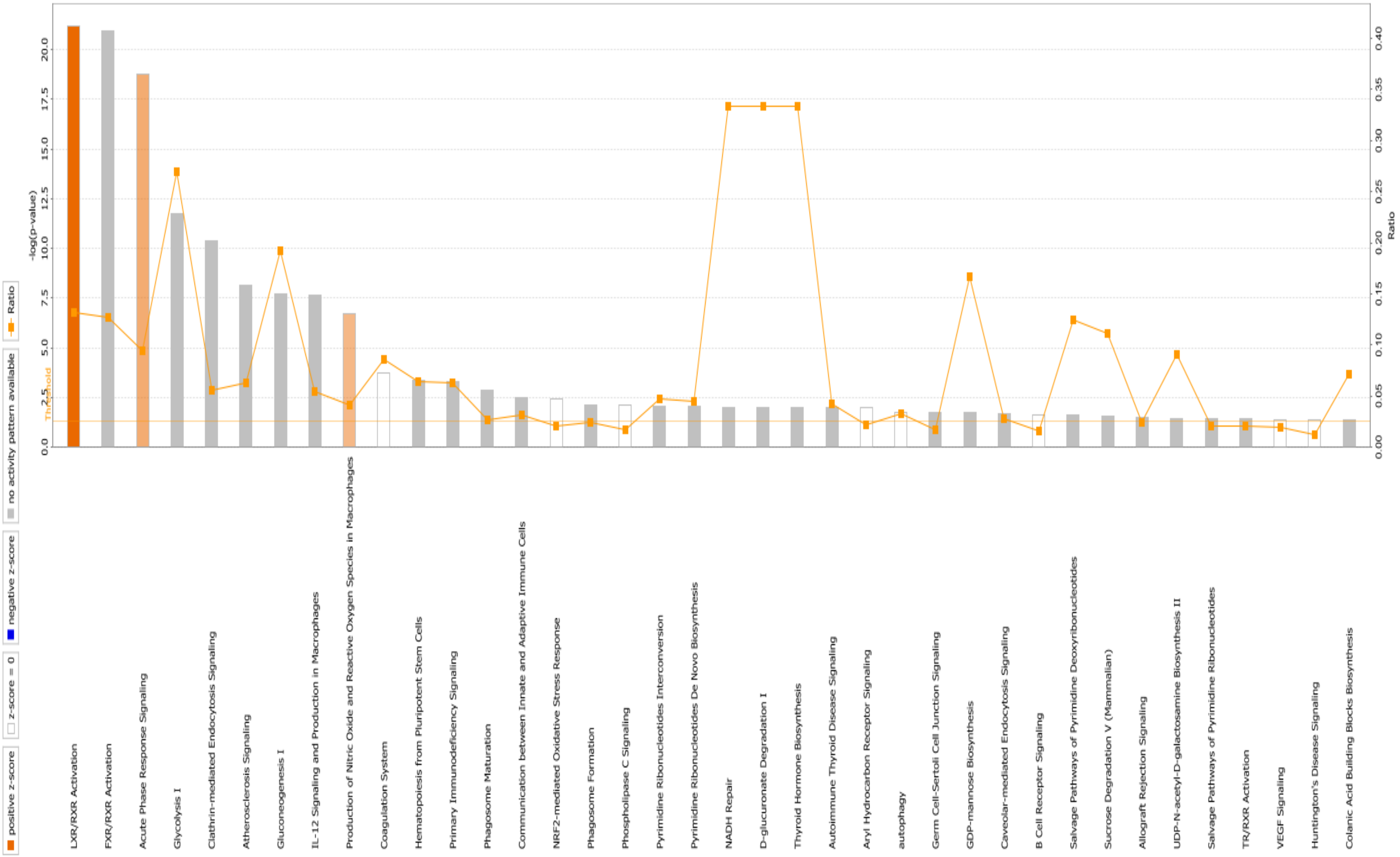

Figure S2. Canonical pathways of soft CL tear proteins. Major canonical pathways represented as bar chart of the differentially expressed tear proteins of the soft CL users. The p-values of the proteins within the each canonical pathway was determined using the Benjamini-Hochberg corrected Fisher's exact test and ratio indicate the number of molecules in the dataset associated with a pathway to the total number of molecules identified in that particular pathway.
